# Complementary Hand Responses Occur in Both Peri- and Extrapersonal Space

**DOI:** 10.1371/journal.pone.0154457

**Published:** 2016-04-27

**Authors:** Tim W. Faber, Michiel van Elk, Kai J. Jonas

**Affiliations:** Department of Psychology, University of Amsterdam, Amsterdam, the Netherlands; Università di Parma, ITALY

## Abstract

Human beings have a strong tendency to imitate. Evidence from motor priming paradigms suggests that people automatically tend to imitate observed actions such as hand gestures by performing mirror-congruent movements (e.g., lifting one’s right finger upon observing a left finger movement; from a mirror perspective). Many observed actions however, do not require mirror-congruent responses but afford complementary (fitting) responses instead (e.g., handing over a cup; shaking hands). Crucially, whereas mirror-congruent responses don't require physical interaction with another person, complementary actions often do. Given that most experiments studying motor priming have used stimuli devoid of contextual information, this space or interaction-dependency of complementary responses has not yet been assessed. To address this issue, we let participants perform a task in which they had to mirror or complement a hand gesture (fist or open hand) performed by an actor depicted either within or outside of reach. In three studies, we observed faster reaction times and less response errors for complementary relative to mirrored hand movements in response to open hand gestures (i.e., ‘hand-shaking’) irrespective of the perceived interpersonal distance of the actor. This complementary effect could not be accounted for by a low-level spatial cueing effect. These results demonstrate that humans have a strong and automatic tendency to respond by performing complementary actions. In addition, our findings underline the limitations of manipulations of space in modulating effects of motor priming and the perception of affordances.

## Introduction

Imitation is a key characteristic of human beings [1]. Copying actions performed by others either intentionally or automatically is however not restricted to copying similar actions (moving your right finger when perceiving a right finger movement) but often requires complementing actions as well (e.g., shaking an extended hand; handing over a cup). While both mirror and complementary actions can be driven by the same social information (e.g., hand gestures), mirror actions do not involve direct interaction while complementary actions often do. In the present study we investigated whether the tendency to imitate or complement others’ actions depends on the opportunity to directly interact with them. In a world in which we are increasingly surrounded by possibilities for virtual interactions (e.g., through virtual reality gaming, Skype, 3D movies etc.) insight in this topic is of high importance. One theoretical possibility is that mirror and complementary actions are a product of automatically detecting the potential for action (affordances) as dispositional properties of the observed person or object [2]. More specifically, information about a person or object properties such as knowing that an extended hand can be complemented with an opposite hand (i.e., handshake) might be sufficient to trigger a complementary response, irrespective of an individual’s opportunity to actually perform this response. This reasoning fits well with evidence from research on object perception, which has shown how people make complementary actions towards objects even when motor properties associated with these objects are task-irrelevant and regardless of an object being physically manipulable or not [3–8].

Recently however, neurophysiological and behavioral studies have demonstrated how perceived distance of objects in space modulates both observation and interaction with objects [9–11]. Whereas this distance modulation has been shown for objects, it has not been shown for social actions (e.g., hand gestures), likely because these cues are primarily used to study imitation for which physical contact is not a prerequisite [12]. Social actions differ from objects given that they can trigger making either a congruent (from a mirror perspective) or incongruent (complementary) response such as imitating finger movements or shaking a person’s hand. We note that this distinction might be confusing given that complementary actions are denoted here as incongruent in terms of mirror perspective but can at the same time be congruent in terms of outcome (i.e., handshaking as a social response outcome) or be anatomically congruent to the observed gesture (e.g., making a right open hand movement upon seeing an actor’s right open hand movement). However, we decided to use this terminology for the sake of consistency with the discussed literature, for a more inclusive definition see [13].

The question we pose is whether the perceived opportunity to interact with another person affects motor priming in response to social actions (i.e., making a fist or an open hand gesture). In five studies we test whether and how responding to hand gestures performed by an actor onscreen, by either making a congruent (mirror) or incongruent (complementary) response is modulated by the depicted distance in space between the actor and observer. Before describing the current research effort, we will summarize research on the effects of motor priming and the modulating role of peripersonal space on these effects.

### Motor priming and space

A large number of studies have demonstrated how observing actions (goal-directed or ‘meaningless’) can activate certain brain areas that are also involved in the performance of these actions (i.e., the so-called mirror neuron regions; see [14–16]). Besides neurophysiological and brain imaging evidence, much support for this visuomotor interaction where perceived actions trigger associated motor programs comes from behavioral response paradigms [6, 12, 17]. Importantly, a number of studies have shown how an observed action maps onto the representation of the identical or same action in the perceiver (i.e., direct matching). For example, people are quicker to perform a finger movement (upwards or downwards) in response to a symbolic cue when concurrently observing a congruent (mirror) finger movement relative to an incongruent movement [12, 18].

Besides examples of direct matching, recent findings have suggested how motor observation can also trigger more functional or complementary responses [19–20]. For example in Sartori, Cavallo, Bucchioni, and Castiello (2012; [7]), participants showed automatic (covert) congruent responses when observing a hand grip towards an object but showed complementary, non-identical responses when observing grip postures that signaled a complementary request. One framework accounting for identical as well as non-identical motor responses to observed actions is the theory of associative sequence learning (ASL; [21–23]). This theory proposes that motor priming is a product of domain general learning mechanisms that account for congruent as well as incongruent priming effects, given that both are a result of the same sensorimotor learning process. Some findings supportive of ASL have shown a facilitation for making incongruent relative to congruent gestures after only a short reverse training for both meaningless gestures (e.g., making an open hand gesture in response to a closing hand) as well as for object directed actions (Heyes, Bird, Johnson, & Haggard, 2005; [24], van Schie, van Waterschoot, & Bekkering, 2008; [25]). Besides creating novel associations [24, 26, 27] other studies have demonstrated how existing contingent stimulus-response associations may underlie motor priming. In Liepelt, Prinz, and Brass (2010; [28]) for example, participants were faster in making congruent (mirror) hand movements towards intransitive hand gestures (i.e., a fist) while for communicative gestures (i.e., an open hand) participants were faster in responding with incongruent relative to congruent hand movements signaling a complementary hand response (i.e., handshaking). These findings corroborate an earlier study by Flach, Press, Badets, and Heyes (2010; [29]) in which the same complementary effect was found for open hand gestures but not for arrows (see also Sartori, Cavallo, Bucchioni, & Castiello, 2011; [8]).

Taken together it seems that differences between direct matching and more functional examples of motor priming are determined by the (minimal) social meaning of the cue and the task context. This fits with the idea that even though direct matching might suffice for low-level motor cues, in interactive settings matching behavior displayed by others is often suboptimal [30]. For example, when somebody throws you a ball or falls down the stairs a complementary response (e.g., catching; helping) is more fitting than copying the perceived behavior (e.g., throwing; falling; [31]). Diverse studies on joint action [32–35] have underlined the importance of interactive settings in producing task-relevant complementary actions. Still, the majority of motor priming tasks do not include information that signals the possibility to interact with social stimuli (real or imagined) and has commonly focused on mirror or complementary actions separately (at least for different types of gestures). One type of information that resolves this is (interpersonal) space, or the opportunity to interact with objects or others if they are within reach (although not all interactive settings require this such as throwing and catching a ball). By manipulating space in a motor priming task we hypothesize that the perceived opportunity to interact should not affect motor imitation, given that motor imitation does not require direct (physical) interaction, but should affect complementary responses to hand gestures (e.g., open hand) which can only be performed when the other person is within direct reach.

Early support for the role of space in motor processing comes from Rizzolatti, Fadiga, Fogassi, and Gallese (1997; [36]) who stated that visual input is discriminated in terms of manipulability as a function of the space around the body within (peripersonal space) or outside of reach (extrapersonal space). For example, Caggiano, Fogassi, Rizzolatti, Thier, and Casile (2009; [9]) found that in monkeys some mirror neurons selectively responded to the observation of object-directed actions performed by an actor in either the peri- or extrapersonal space of the monkey. These space-selective neurons therefore seemed to dissociate between object-directed actions that could be performed immediately (when the object was in the monkey’s peripersonal space) and actions that could not be performed (i.e., when the object was outside of peripersonal space). This seems to suggest that the space around the body is defined by the function it provides for an individual to perceive and manipulate objects that lie within this space [37].

Research using behavioral paradigms has similarly demonstrated how motor priming is modulated by peripersonal space [10, 38]. For example in Costantini, Ambrosini, Tieri, Sinigaglia, and Committeri (2010; [10]), participants were faster to pantomime a correct relative to incorrect grip response towards an object (mug) but only when the object was presented in peri- and not in extrapersonal space. Importantly, the effect of distance was replicated when instead of manipulating distance through onscreen pictures, objects were placed in a physically reachable location [39]. Here, participants were faster to pantomime congruent object-directed actions only when the mug was positioned within physical reaching distance and not when outside of reach.

Taken together, motor system activity in response to perceived objects (social or non-social) seems to be affected by the (peripersonal) space or the perceived opportunity to interact with them. For our current study we chose a simplified behavioral response paradigm to test whether social cues selectively facilitate responding as a function of the perceived opportunity to interact with the other person. We first propose that congruent (mirror) motor responses to intransitive gestures do not require interpersonal contact whereas complementary responses to open hand gestures do. Therefore, presenting gestures in close distance (i.e., within peripersonal space) should only affect motor priming for open hand and not to intransitive gestures. The gesture types and design are primarily based on Liepelt et al. (2010; [28]) in that we presented both fist and open hand gestures and looked at the response time between perceiving the gesture and performing a congruent or incongruent hand gesture. We expected that participants would show faster incongruent (complementary) responses to an open hand in close distance relative to performing a congruent response. When the same open hand was depicted in far distance this complementary advantage was predicted to disappear.

## Study 1

In line with Liepelt et al. (2010; [28]) we instructed participants to mirror hand gestures made by a visual actor onscreen either with the same specular hand (congruent response; actor’s right hand–participant’s left hand) or using the same anatomical hand as the hand displayed (incongruent response; actor’s right hand–participant’s right hand). The actor either made a fist or an open hand gesture with his right or left hand. Similar to the handshaking effect found in Flach et al. (2010; [29]) and Liepelt et al. (2010; [28]) we predicted a complementary effect (faster incongruent relative to congruent responses) for open hand trials but not for trials involving a fist as hand gesture. To investigate whether the complementary effect would change as a function of space we showed the visual co-actor sitting either opposite on the short end (close) of a table or on the long end (far) of a table. With respect to fist gestures we predicted, in line with Liepelt et al. (2010; [28]), an advantage for performing congruent relative to incongruent hand responses irrespective of interpersonal distance whereas for open hand gestures we predicted that participants would be faster at making incongruent responses compared to congruent responses only in close distance.

To control for our experimental distance manipulation, we asked participants to rate the reachability of the actor in the task in both close and far distance trials (see [11]). After the task participants indicated whether it would be possible to touch the hand of the actor sitting across from the short and long end of the table (separately), if they would be positioned opposite to the actor.

### Participants

Forty one participants (mean age = 22; range = 18–34), including 34 females, participated in the experiment in exchange for either course credit or monetary compensation. In total, 36 participants were right-handed as assessed through self-report. All participants signed an informed consent form before participating in the study. The study was approved by the Psychology Department of University of Amsterdam ethics committee (2014-SP-3731).

### Materials

The stimulus material in the study consisted of photographs made in a lab room with an actor sitting behind a table. All possible hand gestures were photographed so that the actor was shown sitting either behind the short or far end of the table. The exit-questionnaire was used to assess if touching hands with the actor sitting across either side of the table was perceived to be possible. In addition, a number of demographic questions were included.

### Procedure and design

The experiment used a 2 x 2 x 2 design with Distance (close vs far), Hand gesture (open hand vs fist) and Hand response (congruent vs incongruent) as within-subject factors. Participants took part in the experiment as the last of a series of three studies. They were seated at a table with a response box placed in front of them at the center of the table. Behind the response box a computer screen was positioned (resolution: 1680 x 1050 pixels; 22-inch diagonal; 60 Hz refresh rate) on which the visual stimuli were displayed. The custom modified response box (Psychology Research Tools Inc., 2012) had three horizontally aligned response keys, and the most right and left key was used as functional response key, respectively, in the experiment.

Participants were instructed to mirror the specific hand gesture (fist vs open hand) displayed by an actor onscreen, by responding with the same specular hand as the hand appearing onscreen (i.e., congruent response). In half of the trials the hand was given a color, which meant participants had to mirror the hand gesture but then using their hand opposite to the displayed hand on the screen (same anatomical hand; incongruent response).

**Fig 1:**
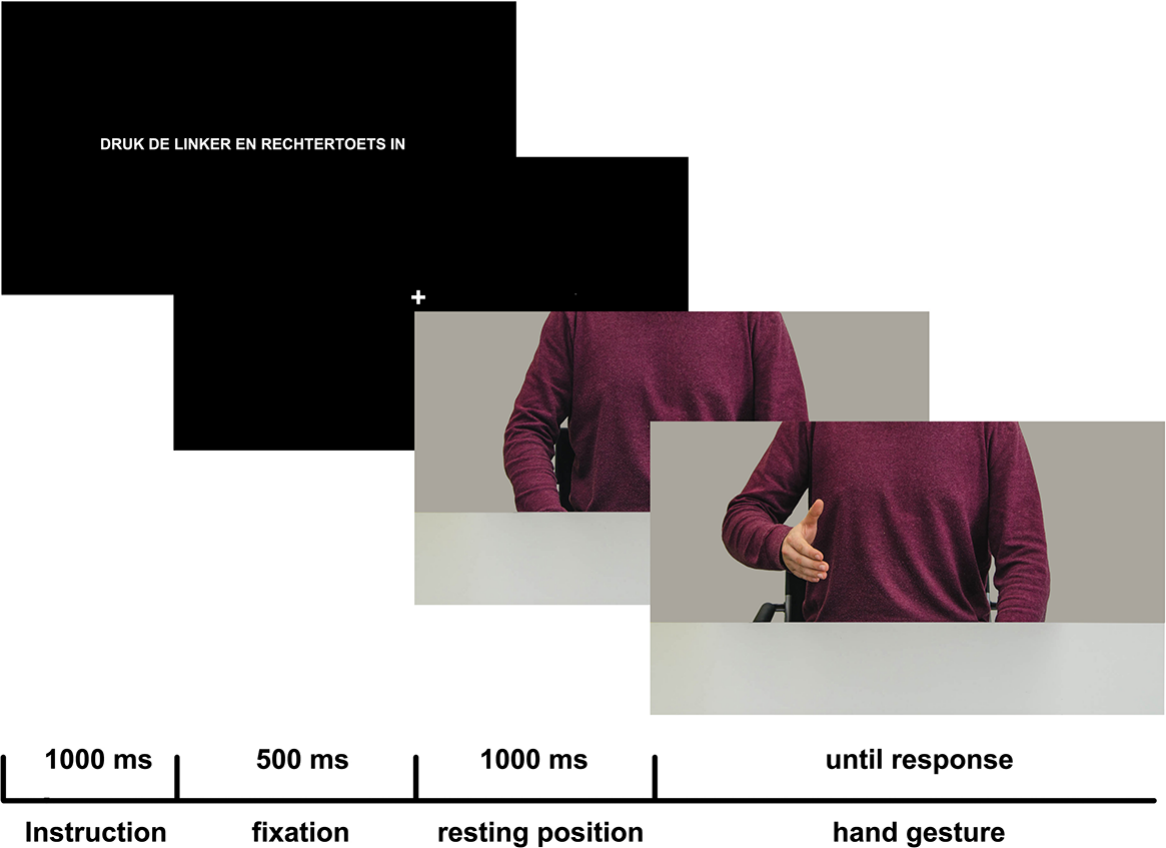
Experimental setup. The general experimental design for the first four studies. The first screen includes the text: “Press and keep pressing the right and left key”. Only non-colored hands are shown here.

**Fig 2:**
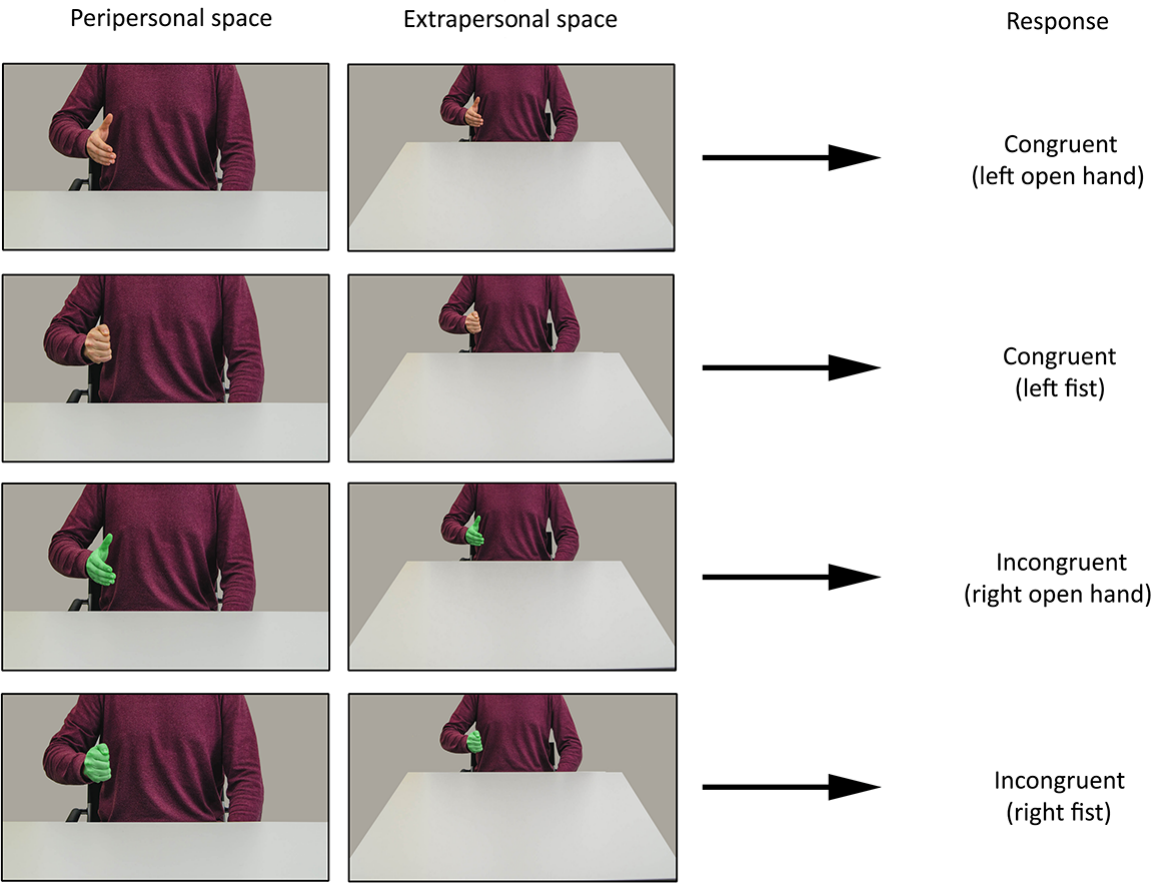
Full design. Full design for the first study. Note: Hands were presented on either side of the screen (left and right), here only hands on the left side are shown. Also, the bottom two rows display the hand gestures including a green (medium opacity) colored layer on top that makes the hand appear green but still sufficiently visible in terms of texture.

After the instruction participants received an additional vocal instruction to make sure the task was clear. Each trial in the main task (see Fig 1) started with a message displayed in the center of the screen for 500 milliseconds (ms) instructing participants to hold the left and right key of the response box pressed with their left and right index finger. When the message disappeared a picture was presented at the center of the screen (1400 x 798 px; consistent with a 32° horizontal visual angle and a viewing distance of 70 cm) depicting a man seated at a table with his hands alongside his body under the table. The man was displayed from the lower part of the neck down, either sitting across the short side (width) of a table (from the perspective of the participant) or the long end of a table for 1000 ms. Following this rest picture a second picture (gesture) was shown in which the actor had lifted his right or left arm and made either a fist or an open hand gesture while keeping the other hand/ arm at resting position. The gesture picture always displayed the same distance position (i.e., short or long end of the table) as the rest picture. In half of the trials a non-colored hand was shown indicating that a congruent response had to be made (see Fig 2). In the other half of the trials the hand was colored green (medium opacity) instructing participants to perform an incongruent hand gesture, mirroring the observed hand gesture using the opposite hand. Response time was calculated from the onset of the gesture picture until participants released the key on the response box in order to perform the hand gesture. In total, 128 trials were presented, in which the presentation of specific trials was fully randomized with respect to distance, hand gesture and hand response. Before the main task participants went through a training session for 28 trials, randomized similar to the main task, to make sure participants were sufficiently acquainted with the task rules. Individual performance was monitored shortly during the training session by the experimenter. During the onscreen instruction it was only shortly mentioned that the depicted actor was sitting at either the short or long end of the table. No specific instructions were provided with regard to the relevance of the spatial distance for the hand response. When finished, participants filled in an exit-questionnaire and were debriefed.

The color cue instructing participants to mirror the perceived hand gesture using their opposite (incongruent) hand was not counterbalanced across participants or conditions (i.e., responding to a color cue with alternating congruent and incongruent hand responses). Although this decision might have affected the results this would have only strengthened rather than weakened the main findings. Given that incongruent responses to open hand or fist gestures require a secondary step (mirror the gesture–use non-corresponding hand) this would have potentially slowed reaction times rather than made it faster (see [25]).

### Data analysis

Response errors were first deleted from the task but used separately to analyze differences in response errors between different trial types. For all trials (for participants combined), 6% (305 trials) of response trials with a wrong response (a mirror response to a green colored hand and a complementary response to a normal colored hand) were deleted. In addition we excluded 1.9% (99 trials) of the total sum of trials in which response times were above or below 2.5 x *SD* from the mean of each individual participant.

### Exit-Questionnaire

In the exit-questionnaire participants were asked whether they could imagine making physical contact (touching hands) with the actor opposite of the table either in the short or far distance setting in order to assess the perceived opportunity to interact. One participant did not complete the exit-questionnaire and was removed before analyzing the data. For the remaining participants, in total 45% (18) imagined it was possible to touch the hand of the person in the short setting while for the far setting only 12.5% (5) imagined this to be possible. Besides ratings of reachability the exit-questionnaire included demographic questions (sex and age) and a question concerning handedness (“Are you left or right-handed?”).

### Results

**Fig 3:**
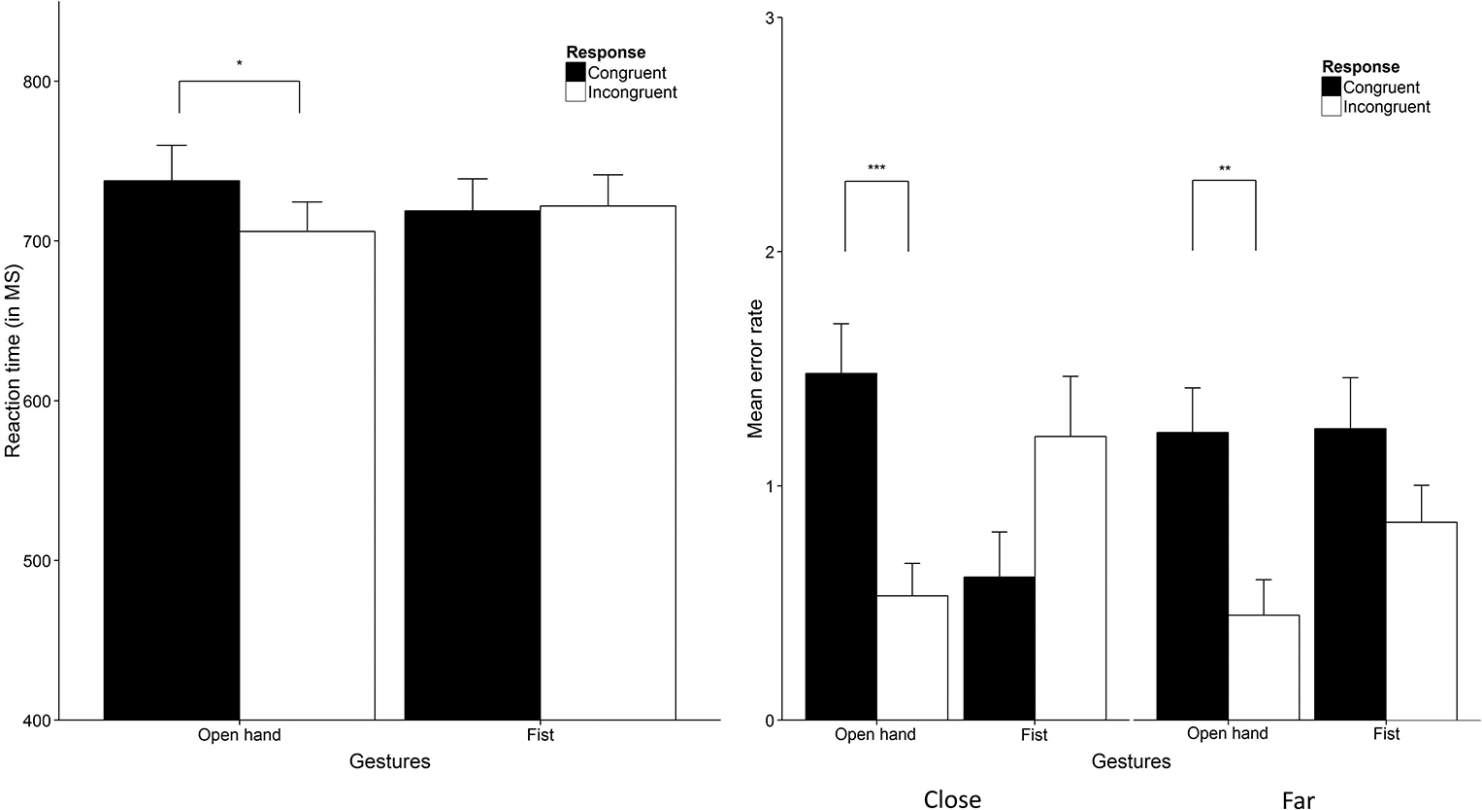
Response data Study 1. The RT results (Left) show reaction time in milliseconds averaged over distance (close and far) trial. The right graph shows error responses for both close (left) and far distance (right) trials. Error bars are 1 x SE. * = *p* < .05, ** = *p* < .01, *** = *p* < .001. All figures were created using ggplot2 [40].

To analyze response time data we ran a 2 x 2 x 2 repeated measures analysis with Distance (close vs far), Hand gesture (fist vs open hand) and Hand response (congruent vs incongruent) as within-subject factors (for RT and error data, see Fig 3). The analysis yielded a non-significant main effect for Distance as well as a non-significant main effect for both Hand gesture and Hand response (all *F*s < 1). Also, no interaction between Distance and Hand gesture was found (*F* < 1). The interaction between Distance and Hand response did not reach statistical significance either, *F*(1, 39) = 3.75, *p* = .060, η^2^_p_ = .09. We did however, find a significant interaction between Hand gesture and Hand response, *F*(1, 39) = 16.78, *p* < .001, η^2^_p_ = .30. Breaking down this interaction yielded a non-significant difference in response latencies between congruent and incongruent responses to fist gesture trials, *t*(39) = -1.41, *p* = .167. Response latencies were faster for incongruent (*M* = 706, *SD* = 117) compared to congruent (*M* = 738, *SD* = 140) responses to open hand trials irrespective of distance, *t*(39) = 2.63, *p* = .012, *d* = 0.48 (*d*_z_ throughout). The three-way interaction between Distance, Hand gesture and Hand response was not significant (*F*(1, 39) = 0.02, *p* = .691), contradicting our main hypothesis.

One option is that the specific features of the cue (e.g., type of gesture; type of distance) require different processing times so that the potential modulation of distance on the complementary effect is found only in slower responses rather than faster. To investigate this, we segmented response time latencies in two time-bins focusing separately on the fastest and slowest responses. Running a repeated measures analysis with Distance, Hand Gesture and Hand response revealed a Hand gesture by Hand response interaction for the fastest responses (time-bin 1), *F*(1, 39) = 16.74, *p* < .001, η^2^_p_ = .30, as well as for the slowest responses (time-bin 2), *F*(1, 39) = 14.20, *p* = .001, η^2^_p_ = .27. For both time-bins no interaction with Distance, Hand gesture and Hand response was found (both *F*s < 1).

For the response errors (e.g., a congruent response to a green colored hand) we performed the same 2 x 2 x 2 repeated measures analysis as for the RT data based on the number of response errors per trial type. We found no main effects for Distance and Hand gesture (both *F*s < 1) but we did find a main effect for Hand response, *F*(1, 39) = 7.96, *p* = .007, η^2^_p_ = .17. Also, a two-way interaction was found between Distance and Hand response, *F*(1, 39) = 4.12, *p* = .049, η^2^_p_ = .10 as well as between Hand gesture and Hand response, *F*(1, 39) = 11.83, *p* = .001, η^2^_p_ = .23. However, these effects were all qualified by a three-way interaction between Distance, Hand gesture and Hand response, *F*(1, 39) = 7.75, *p* = .008, η^2^_p_ = .17. We performed follow-up tests for the three-way interaction (corrected for multiple comparisons using a Bonferroni correction). A higher error rate was found for congruent (*M* = 1.48, *SD* = 1.34) relative to incongruent responses (*M* = 0.53, *SD* = 0.88) in open hand trials, *t*(39) = 4.05, *p* < .001, *d* = 0.64, whereas this difference was not found for fist trials (*p* = .068). In far distance trials we found the same pattern as indicated by a higher error rate for congruent (*M* = 1.23, *SD* = 1.21) compared to incongruent responses (*M* = 0.45, *SD* = 0.96) in open hand trials, *t*(39) = 3.54, *p* = .001, *d* = 0.57, while no difference was found for fist trials.

### Discussion

In line with Liepelt et al. (2010; [28]) we found a complementary effect (i.e., faster incongruent relative to congruent responses) for open hand gestures. Contrary to our hypothesis the results indicate that this effect is found irrespective of spatial distance. Furthermore, the error pattern suggests that the tendency to make a complementary response to open hand gestures, as indicative of a higher error rate for congruent responses to open hand gestures, was evident in both close and far distance trials. Furthermore, no congruency effect (faster responses in congruent compared to incongruent trials) was found for fist gestures contrary to Liepelt et al. (2010; [28]). We discuss further issues with respect to this finding as well as the unexpected variability of reachability ratings in the re-analysis paragraph following Study 5.

One possibility for the absence of an effect of distance is that the close versus far distance images were not well designed to convey visual differences in peripersonal space given that both close and far distance images literally appeared at an equal physical distance from the participant. Also, perhaps the smaller spatial distance between the gesture position and the center of the screen in the far distance trials may have differentially affected hand responses. To control for visual differences (of the actor and response hand) and to replicate the task using a stronger manipulation of peripersonal space we ran a second study in which we used the same stimuli but with a glass screen placed on the table in front of the actor, providing a measure of functional rather than physical space (see Costantini et al., 2010; [10]). Given that peripersonal space requires that people or objects are able to directly interact, the placement of a screen would obstruct such an interaction. Somewhat in line with Heed, Habets, Sebanz, and Knoblich (2010; [41]) if an actor is seated in peripersonal space but not engaged in all aspects of the task this is not sufficient to foster interpersonal coordination. Therefore, if an actor is seated in peripersonal space but is unable to interact with the participant this should interfere with producing complementary motor responses.

## Study 2

**Fig 4:**
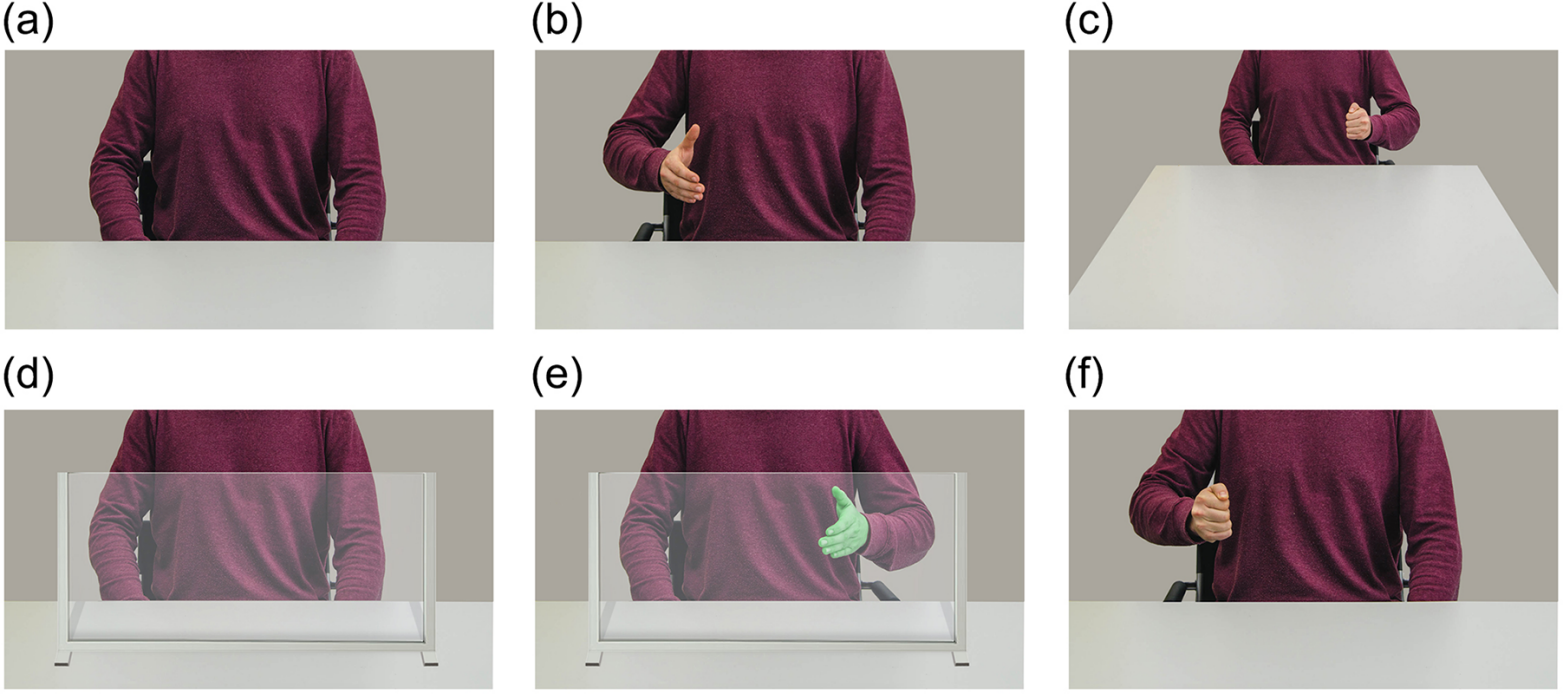
Sample images. Sample images from Study 1, 2 and 4. (a) Resting position close (b) Open hand left close (c) Fist right far (d) Resting position frame (e) Open hand green right frame (f) Fist left close.

Study 2 only differed from Study 1 in terms of the stimulus set. As in the previous study there was a 2 x 2 x 2 within subjects design but here with Frame (glass frame vs no frame), Hand gesture (open hand vs fist) and Hand response (congruent vs incongruent) as within-subject factors. We used the same stimuli from the previous study depicting hand movements from the close distance trials as no frame trials. In the glass frame trials a glass frame was shown on the table in front of the actor onscreen (see Fig 4). This was done for both the gesture and the rest trials.

### Participants

In this study, 30 participants took part including 21 Females with a mean age of 23 (range 18–30). Twenty-seven participants were right-handed. All participants took part in the study in exchange for course credit or monetary compensation. All participants signed an informed consent form before participating in the study. The study was approved by the Psychology Department of the University of Amsterdam ethics committee (2014-SP-3731).

### Materials and Procedure

For the stimuli, we took the no frame trials and edited, using Photoshop (version CS6; Adobe Systems, 2012) a glass frame on the table in front of the actor. The specific glass frame was chosen from a pretest in order to select a screen that most people would rate as clearly obstructing interpersonal contact. The instructions, setup and task presentation was identical to the first experiment. In total 128 trials were presented additional to a practice run including 28 trials, both fully randomized with respect to frame, hand gesture and hand response.

### Data analysis

In the total number of trials, 6.9% (266) incorrect responses were made which were removed before performing the analysis. Furthermore, we excluded 2% (76) of response trials where the reaction time was above or below 2.5 x *SD* each participant’s mean. Additionally, we excluded response data from one participant who was not a native speaker and had trouble understanding the task instructions.

### Exit-questionnaire

As in the previous study, we asked participants to indicate whether they thought it was possible to make physical contact with the depicted actor on the screen in the no frame and glass frame trials. Of all participants, 51.7% (15) indicated that they thought it would be possible to make physical contact in the no frame trials while all participants thought this would not be possible in the glass frame trials.

### Results

**Fig 5:**
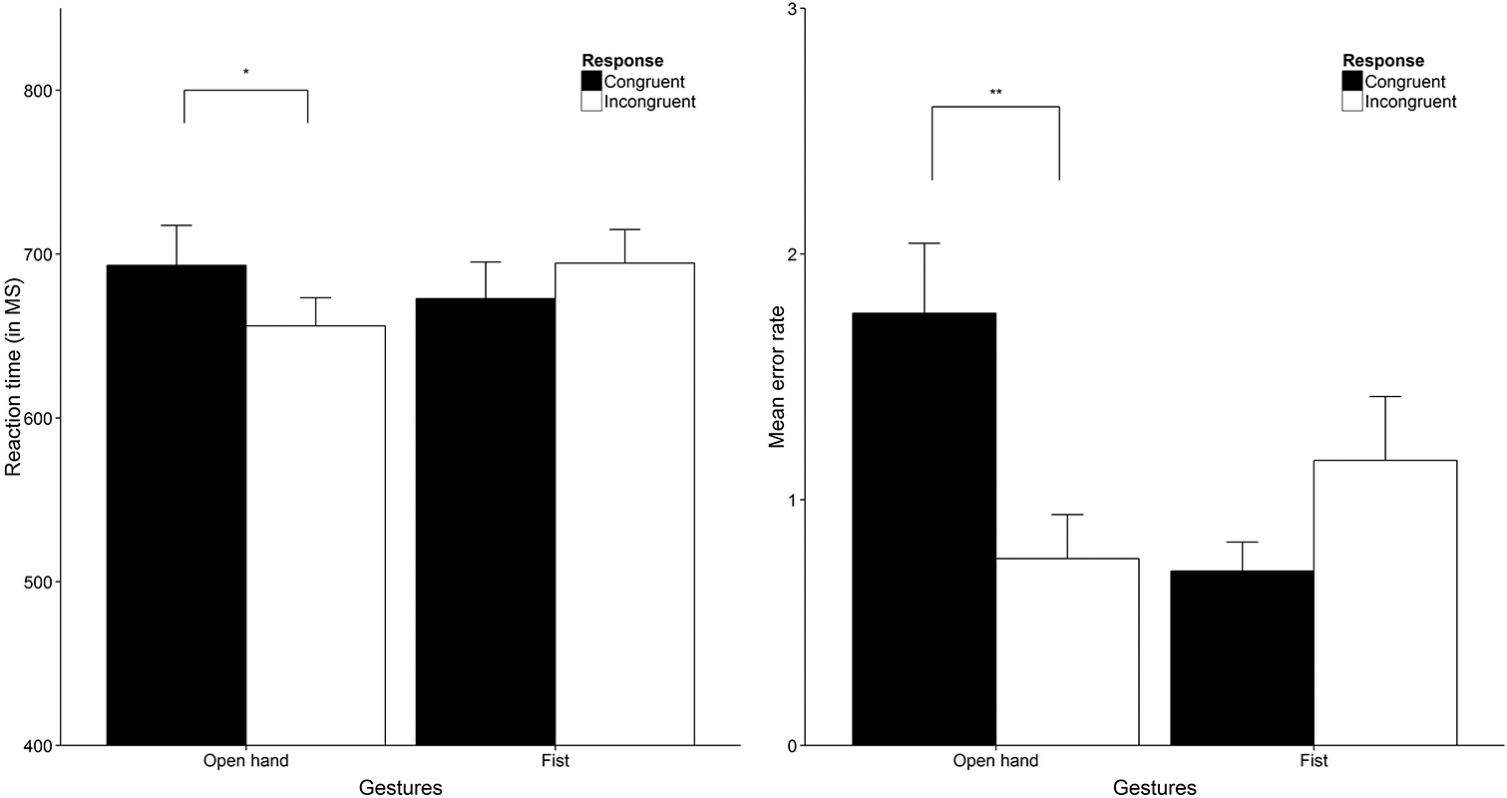
Response data Study 2. The RT results (Left) show reaction time in milliseconds averaged over distance (close and far) trials. The right graph shows mean error responses averaged over distance trials (close and far). Error bars are 1 x SE. * = *p* < .05, ** = *p* < .01, *** = *p* < .001.

For the main analysis we ran a 2 x 2 x 2 repeated measures analysis with Frame (glass frame vs no frame), Hand gesture (open hand vs fist) and Hand response (congruent vs incongruent) as within-subject factors (for RT and error data see Fig 5). First, the analysis showed no significant main effects for Frame, Hand gesture or Hand response (all *F*s < 1). Also, no hypothesized three-way interaction effect was found between Frame, Hand gesture and Hand response (*F*(1, 28) = 1.13, *p* = .297) nor a significant interaction between Frame and Hand response (*F* < 1). We did find an interaction between Frame and Hand gesture, *F*(1, 28) = 6.88, *p* = .014, η^2^_p_ = .20 as well as an interaction between Hand gesture and Hand response, *F*(1, 28) = 17.65, *p* < .001, η^2^_p_ = .39. For the Frame by Hand gesture interaction, we found that participants responded faster to fist gestures in the no frame trials (*M* = 676, *SD* = 108) relative to the glass frame trials (*M* = 691, *SD* = 115) irrespective of the type of hand response made, *t*(28) = -2.51, *p* = .018, *d* = -0.47. No difference was found in response times for open hand trials averaged over hand response type between glass frame and no frame trials. To obtain simple main effects for the Hand gesture by Hand response interaction, we looked at mean response times averaged over levels of Frame. Results showed that participants made faster incongruent responses to an open hand gesture (*M* = 656, *SD* = 92) compared to congruent responses (*M* = 693, *SD* = 131), *t*(28) = 2.45, *p* = .021, *d* = 0.45. For trials where the actor showed a fist gesture, there was no significant difference in response times between incongruent and congruent hand responses, *t*(28) = -1.73, *p* = .095.

For the response error data we found a main effect for Hand gesture, *F*(1, 28) = 5.05, *p* = .033, η^2^_p_ = 0.15 which was qualified by a significant interaction between Hand gesture and Hand response, *F*(1, 28) = 15.25, *p* = .001, η^2^_p_ = .35. Simple main effects indicated that more errors were made in congruent (*M* = 1.76, *SD* = 1.53) compared to incongruent trials (*M* = 0.76, *SD* = 0.97) in response to open hand gestures irrespective of the frame being present or not, *t*(28) = 2.92, *p* = .007, *d* = 0.54. No difference was found in the errors made in response to trials including a fist as hand gesture. No effects of distance on the error rates were observed.

### Discussion

The results were in line with the first study in that incongruent responses to open hand gestures were faster than congruent responses. Moreover, the findings did not show an effect of space, here manipulated with a frame positioned in front of the actor. The results further support the idea that peripersonal space, by either increasing distance or obstructing physical contact, does not affect the performance of complementary hand gestures.

Furthermore, in line with Study 1, the response error data suggest that participants were more inclined to perform incongruent responses in response to open hand trials when congruent responses were required. Although the results provide additional support for the space independence of the complementary effect it might still be the case that the type of stimulus environment does not accurately reflect the setting it is designed to imply (i.e., participants always responded to a 2D stimulus with which no ‘real’ interaction was possible in the first place, thereby rendering our space manipulation mute). Increasing the ecological validity by increasing the realism of the stimuli might be one way to improve the current design.

## Study 3

As suggested, it could be that the type of images used so far was not fit to convey differences in perceived physical or functional space. Therefore, in the third study we used the exact same task as in the first study but then presented the images on a pair of 3D glasses to enhance the experience of interpersonal space. A similar manipulation was used in Costantini, Ambrosini, Scorolli, and Borghi (2011; [42]) who let participants categorize words that specified motor responses to objects that were presented at different points in space while wearing 3D glasses.

### Participants

Twenty-one participants took part in the third study including 13 Females with a mean age of 22 years old (range 19–27). In total, 19 out of 21 participants were right-handed. All participants received either course credit or monetary compensation and signed an informed consent form before participating in the study. The study was approved by the Psychology Department of the University of Amsterdam ethics committee (2014-SP-3880).

### Materials and procedure

A new set of stereoscopic stimuli was created that matched the stimulus set used in the first study. These pictures were presented using an Oculus Rift head-mounted display (version DKII; Oculus VR, 2014). The same response box and procedure was used as in the previous two studies. Additionally, we added to the onscreen instruction that the actor seated across from the short end of the table was within reach whereas the actor seated across from the far side of the table was outside of reach. This was done in order to stress the differences in interpersonal distance in the images. Also, for the practice part participants went through 20 practice trials without the 3D device followed by 20 trials with the device. The main task included 128 trials, randomized with respect to distance, hand gesture and hand response. Finally, we removed the onscreen instruction in the task that indicated to press and keep pressing the right and left key at the start of each trial. The reason to do this was that accommodating to a stereoscopic display of instructions was quite unpleasant for the eyes [43]. Based on the response error data we saw that this change did not increase the total number of response errors (3.3% versus 6% in Study 1 and 6.9% in Study 2).

### Data analysis

For all recorded trials, 3.3% of the trials (89) were removed due to response errors. Also, 2.5% of the trials (55) were removed for which the response latency was above or below 2.5 x *SD* the mean of the participant.

### Exit-questionnaire

In line with the earlier studies we found that still 42.9% (9) of the participants thought it would be impossible to make physical contact with the depicted actor in close distance. All participants thought it would be impossible to do this in the far distance trials.

### Results

**Fig 6:**
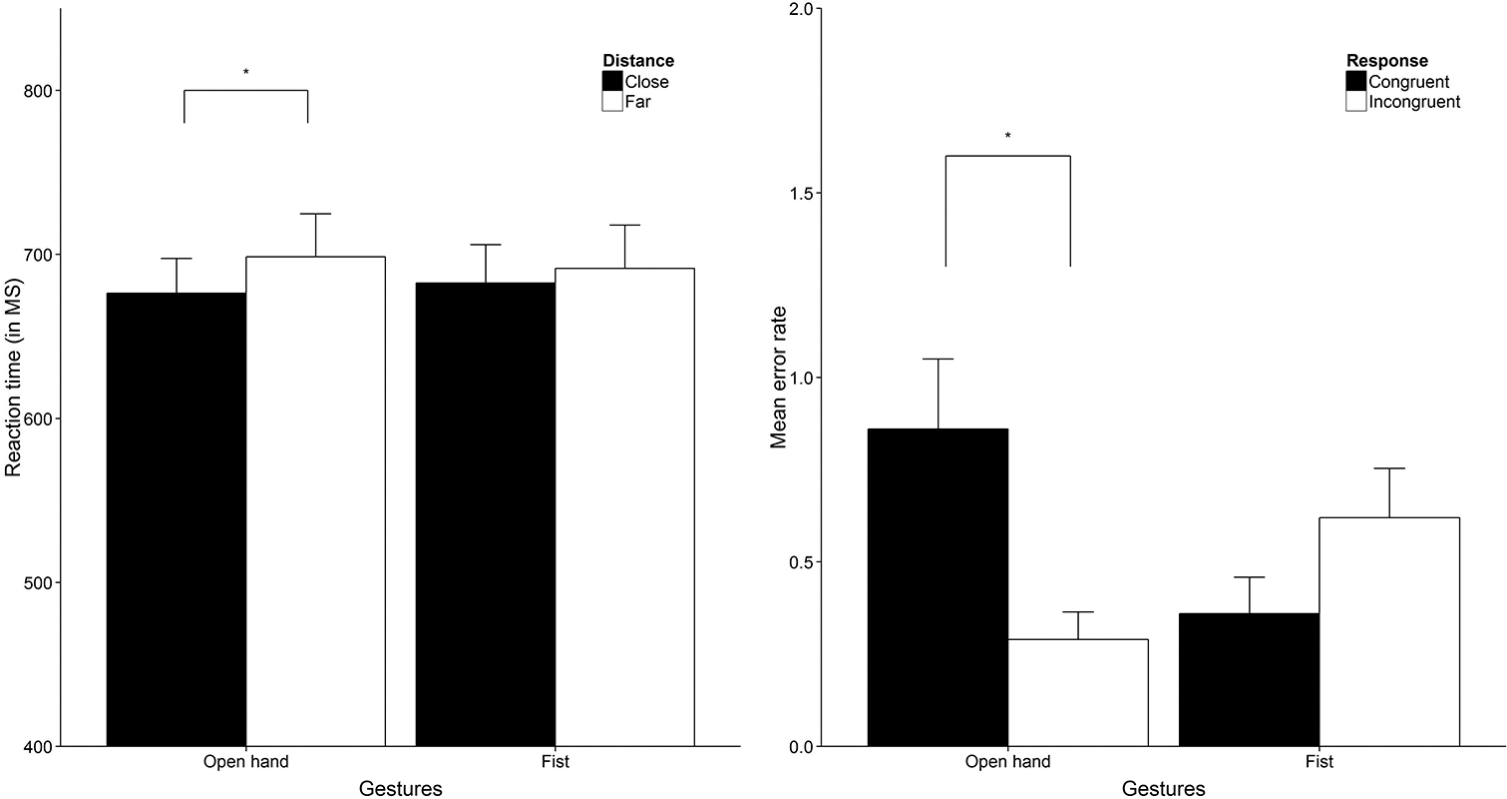
Response data Study 3. The RT results (Left) show reaction time in milliseconds averaged over Hand response (congruent and incongruent). The right graph shows mean error responses averaged over distance (close and far). Error bars are 1 x SE. * = *p* < .05, ** = *p* < .01, *** = *p* < .001.

For the main analysis we conducted a 2 x 2 x 2 repeated measures analysis with Distance (close vs far), Hand gesture (fist vs open hand) and Hand response (congruent vs incongruent) as within-subject factors (see Fig 6). We only found a significant Distance by Hand gesture interaction irrespective of the type of hand response made, *F*(1, 20) = 5.14, *p* = .035, η^2^_p_ = .20. All other main effects and interaction effects including the three-way interaction between Distance, Hand gesture and Hand response yielded non-significant results. Looking at the mean response times per level of hand gesture we found that responding to open hand gestures, irrespective of the type of response, was faster in close (*M* = 676, *SD* = 97) relative to far distance trials (*M* = 698, *SD* = 121), *t*(20) = -2.30, *p* = .032, *d* = -0.50. No difference was found for trials where a fist gesture was shown.

Results from the response error data showed a significant Hand gesture by Hand response interaction, *F*(1, 20) = 9.83, *p* = .005, η^2^_p_ = .33 while no other main or interaction effects reached statistical significance. Simple main effects indicated that more errors were made in congruent (*M* = 0.86, *SD* = 0.87) relative to incongruent response trials (*M* = 0.29, *SD* = 0.34) for open hand gestures irrespective of distance, *t*(20) = 2.61, *p* = .017, *d* = 0.57. No difference was found in the error rate for fist trials and no effects of distance were observed.

### Discussion

The results of the third study showed an effect of distance as indicated by faster responses to open hand gestures in the close relative to far distance trials irrespective of the type of hand response. Although participants’ own hand responses were not visible during the task, thereby eliminating visuomotor feedback, the pattern of response errors matched that of the previous studies suggesting that even though participants could not see their hands complementary responses were facilitated for open hand gestures. The results indicate that motor priming affected hand responses irrespective of the specific outcome (mirror or complementary) as a function of space. Potentially, the effect of space on reaction times might therefore be related to differences in the stimulus set given that images depicted in the 3D glasses appear larger in close distance relative to the onscreen images.

In all studies presented so far, peripersonal space, manipulated by either changing the visual distance or physical obstruction between the participant and actor (Study 2), was always response-irrelevant. In terms of showing an automatic effect of distance on motor priming this is a strength of the design. However when responding to cues in their environment, people might not automatically take social distance information into account. Previous studies regarding the activation of motor affordances have shown that motor representations in response to objects or observed actions for instance, are only activated when these features are made task-relevant [44–45]. By explicitly instructing participants to attend to differences in interpersonal space, it can therefore be investigated if motor priming in response to hand gestures is modulated as a function of distance when perceived distance is made task-relevant. This was the main aim of the fourth study.

## Study 4

In the fourth study we used the same design and setup as in Study 1 but a blocked design was introduced where participants had to respond to hand gestures only when the actor was seated either in close or far distance depending on the block type. The study was approved by the Psychology Department of the University of Amsterdam ethics committee (2014-SP-3880).

### Participants

In the fourth study, 20 participants took part including 12 Females with a mean age of 20 years old (range = 18–26). Thirteen participants were right-handed and three left-handed. Data for handedness for 4 remaining participants was mistakenly not recorded. All participants received course credit or monetary compensation for participating in the study and signed an informed consent form prior to participation.

### Materials and procedure

The same stimuli were used as in Study 1. After a practice round of 20 trials, similar to the first study, participants were told that the main task consisted of 4 blocks, each including 40 trials, where they had to respond to hand gestures only when the actor was positioned across from the short or far end of the table depending on the block instruction. Two block orders where used where far and close distance instructions were alternated starting with either a far or close distance block (i.e., ABAB or BABA). During go-trials (80% of the trials in each block) participants had to respond as instructed in the practice trials (i.e., mirror the hand gesture with either a congruent or incongruent response), during no-go trials (20% of the trials in each block) participants were asked to keep their fingers on the buttons. For example, in a far distance block participants responded to 32 trials depicting an actor seated in far distance while withholding responses in 8 trials where the actor was seated in close distance. Wrong responses on no-go trials were not analyzed. In total, 160 trials were presented in 4 blocks, in which trials were randomly presented with respect to distance, hand gesture and hand response. In addition we changed part of a sentence in the introduction to explicitly state that participants had to imagine sitting at a table with the actor sitting across from them on the other side. Also, as in the third study, we stated that the actor would be sitting within reach for the short distance trials and outside reach in the far distance trials.

### Data analysis

From the total sum of trials, 3.7% (118) of trials were removed due to response errors. Additionally, 0.7% (21) of all trials were removed for which response latencies were either above or below 2.5 x *SD* the individual participant mean.

### Exit-questionnaire

Out of 20 participants, 65% (13) thought it was possible to touch the hand of the actor in the close distance trials while only 15% (3) thought it was possible in the far distance trials.

### Results

**Fig 7:**
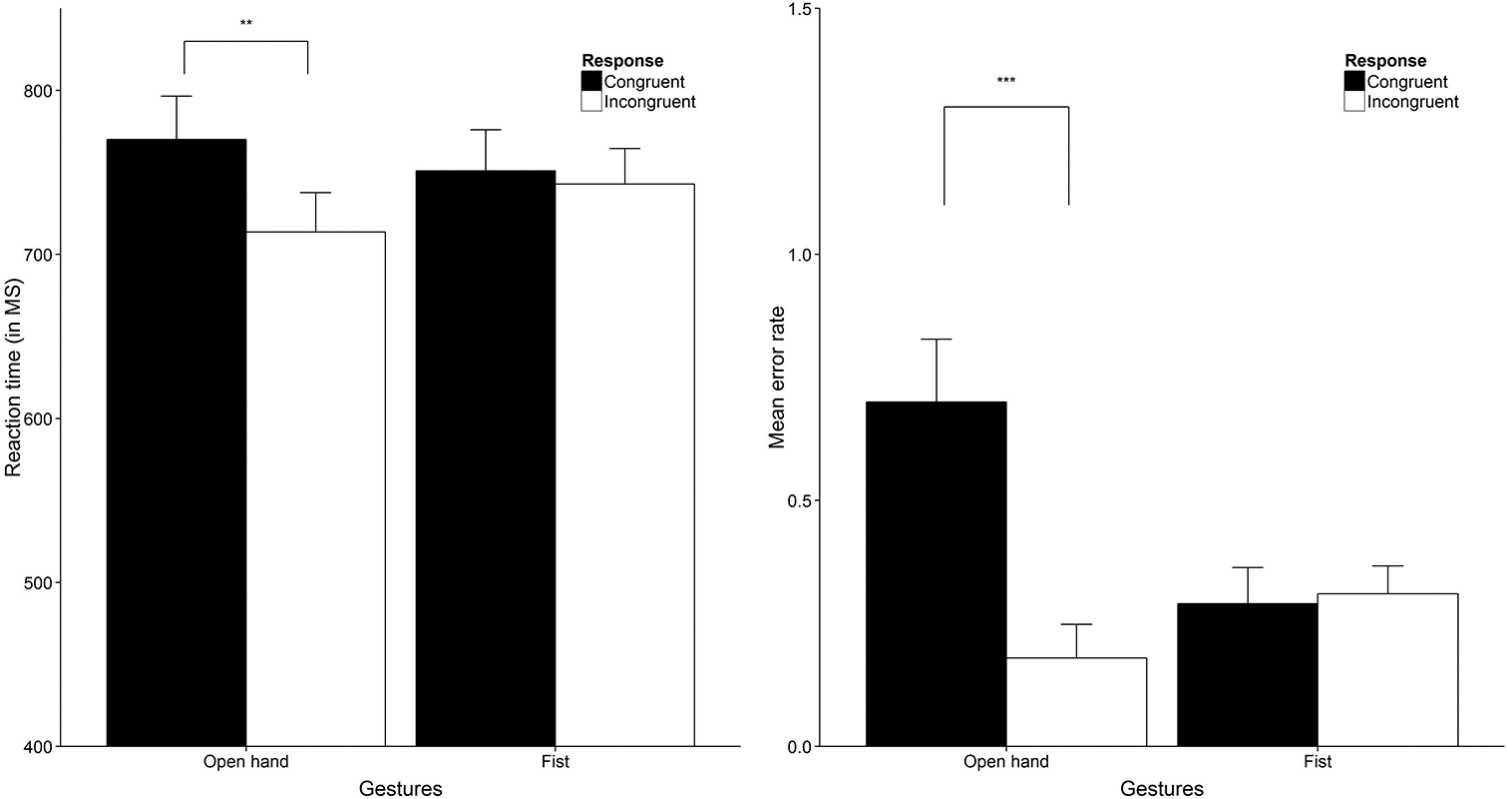
Response data Study 4. The RT results (Left) show reaction time in milliseconds averaged over response type (congruent and incongruent). The right graph shows mean error responses averaged over distance trials (close and far). Error bars are 1 x SE. * = *p* < .05, ** = *p* < .01, *** = *p* < .001.

First of all we checked if block order had any influence on the full within-subjects analysis. Neither a main effect for block order nor an interaction effect between block order and within factors turned out significant (all *p*s>.05). We first collapsed reaction time data over blocks and ran a 2 x 2 x 2 repeated measures analysis with Distance (close vs far), Hand gesture (fist vs open hand) and Hand response (congruent vs incongruent) as within-subject factors (see Fig 7). None of the main effects were significant (all *p*s > .05) nor the hypothesized three-way interaction (*F*(1, 19) = 1.03, *p* = .322), only the interaction between Hand gesture and Hand response yielded a significant effect, *F*(1, 19) = 16.20, *p* = .001 η^2^_p_ = .46. Simple effects indicated that reaction times for congruent responses were slower (*M* = 770, *SD* = 117) compared to incongruent responses to open hand gestures (*M* = 714, *SD* = 108) irrespective of distance, *t*(19) = 3.57, *p* = .002, *d* = .80. There was no difference between congruent and incongruent responses to fist gestures (*p* = .543).

With respect to response errors we found a main effect for Hand response, *F*(1, 19) = 8.44, *p* = .009, η^2^_p_ = .31, indicating that more errors were made in congruent (*M* = 0.49, *SD* = 0.45) relative to incongruent response trials (*M* = 0.24, *SD* = 0.28). More importantly, a two-way interaction was found between Hand gesture and Hand response similar to the previous studies, *F*(1, 19) = 21.09, *p* < .001, η^2^_p_ = .53. Looking at the simple effects we found that participants made more errors when cued to make a congruent (*M* = 0.70, *SD* = 0.57) relative to incongruent response (*M* = 0.18, *SD* = 0.30) in open hand trials, *t*(19) = 4.47, *p* < .001, *d* = 1.00. No difference was found between fist trials where a congruent or incongruent gesture was the correct response (*p* = .785).

### Discussion

The results from the fourth study corroborate the findings of Study 1 and 2 both in terms of reaction time latencies and response errors. Interestingly, making distance task-relevant did not reveal an effect of distance on complementary effects for open hand gestures.

Although the present experimental paradigm consisting of congruent and incongruent hand responses to open hand gestures has been used previously, it could well be that the so-called social complementary hand shaking effect is actually driven by low-level features of the stimulus. For example, one option is that the open hand was interpreted as a directional cue pointing towards the participant’s incongruent relative to congruent response hand, rather than as a social action facilitating a complementary hand response. On this account, the priming effects observed would be a mere consequence of spatial stimulus features rather than reflecting social affordance effects. In favor of this notion, hand responses in a spatial cueing task have been found to be slower if a hand is presented in the center of the screen with a finger pointing away rather than towards the target position of the hand response [46]. This spatial or directional cueing account would fit well with the absence of an effect of distance on motor priming across the four studies that we conducted–as spatial cues are expected to affect motor responses irrespective of the distance at which they are presented (as long as they cue the same relative target location; see [47]). Previous studies have aimed to control for the potential confound that spatial stimulus features actually underlie the effects of observed hand gestures on behavior (see [29, 48]), but these studies have been primarily controlled for spatial confounds in imitative rather than complementary responses or, in the case of Flach et al. (2010; [29]), have replaced hand gestures by arrows rather than hand gestures that form spatial (or directional) cues. The fifth study was done to control for a general cueing effect produced by open hand gestures, which would provide an alternative and more low-level account of the effects observed in the first four studies.

## Study 5

In this study a new set of images was produced in which an actor was displayed using his hand to point with an index finger along the same direction as the open hand pictures used in the first two studies. Given that a pointing finger does not represent a communicative gesture that requires interpersonal contact, this gesture allowed us to control for directional cueing effects produced by open hand trials in the previous studies. Given that our main goal was to see whether the pointing hand would show a directional effect that could replace the complementary effect and not the effect of space on processing the pointing hand, we only looked at hand gestures depicted in short distance.

### Participants

For this study, 25 participants were recruited including 14 Females with a mean age of 23 years (range = 19–47). Nineteen participants were right-handed. All participants received course credit or monetary compensation in return for their participation in the experiment and signed an informed consent form before participating in the study. The study was approved by the Psychology Department of the University of Amsterdam ethics committee (2014-SP-3880).

### Materials and procedure

**Fig 8:**
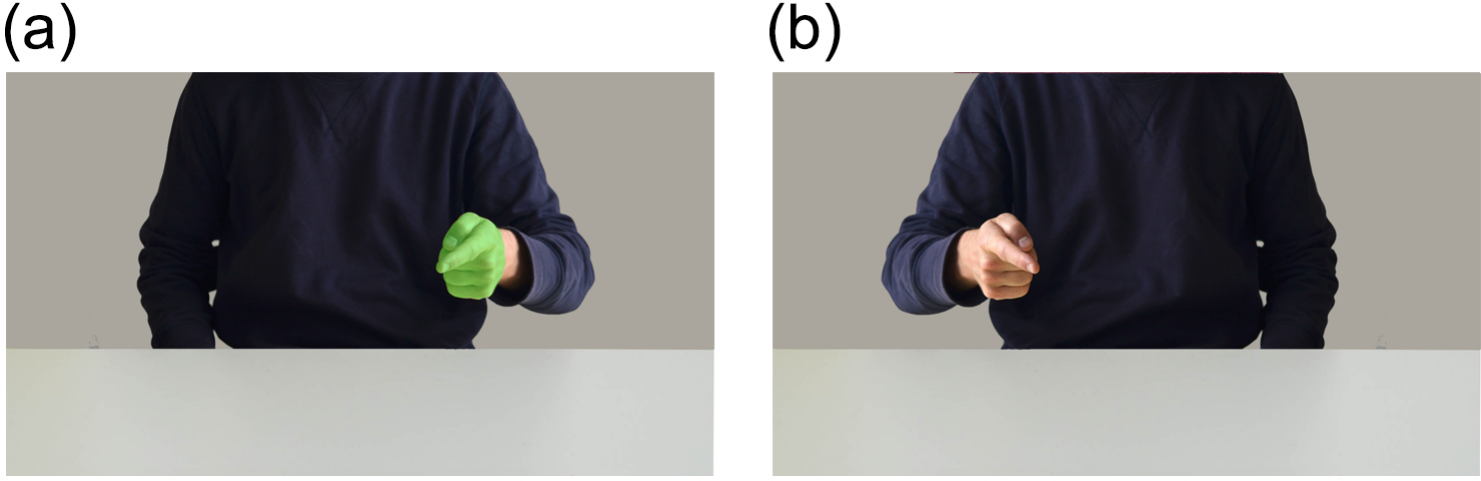
Sample images Study 5. Sample images include (a) Pointing hand green right (b) pointing hand left.

New photographs were made of an actor making either a fist or pointing hand gesture using both the left and right hand. The body and hand gestures were cut out and pasted in the original pictures created in Study 1 (see Fig 8). Only the short distance trials were used for this study, so the introduction was adapted to remove information about the distance at which the actor was positioned. The procedure was the same as in the first study, participants were instructed to always mirror the perceived hand gesture (pointing for point hand gestures–fist for fist gestures) but use their opposite (same anatomical) hand when a green colored hand was observed.

### Data analysis

Two participants were excluded for which no correct responses were recorded for at least one response category. Based on the remaining data, we first removed 2.2% (41) of the total sum of trials for which the reaction time was 2.5 x *SD* above and below the individual participant mean. Furthermore, 10% (184) of trials were removed due to response errors.

### Exit-questionnaire

Given that we only used close distance trials in this study we had participants only rate the close distance trials in terms of potential reachability. In total, only 21.7% (5) of participants indicated it would be possible to touch the hand (pointing hand gesture) of the actor opposite from the table. In addition we asked whether participants felt they wanted to touch the hand of the actor. Only 10% (2) of participants felt they wanted to do this.

### Results

**Fig 9:**
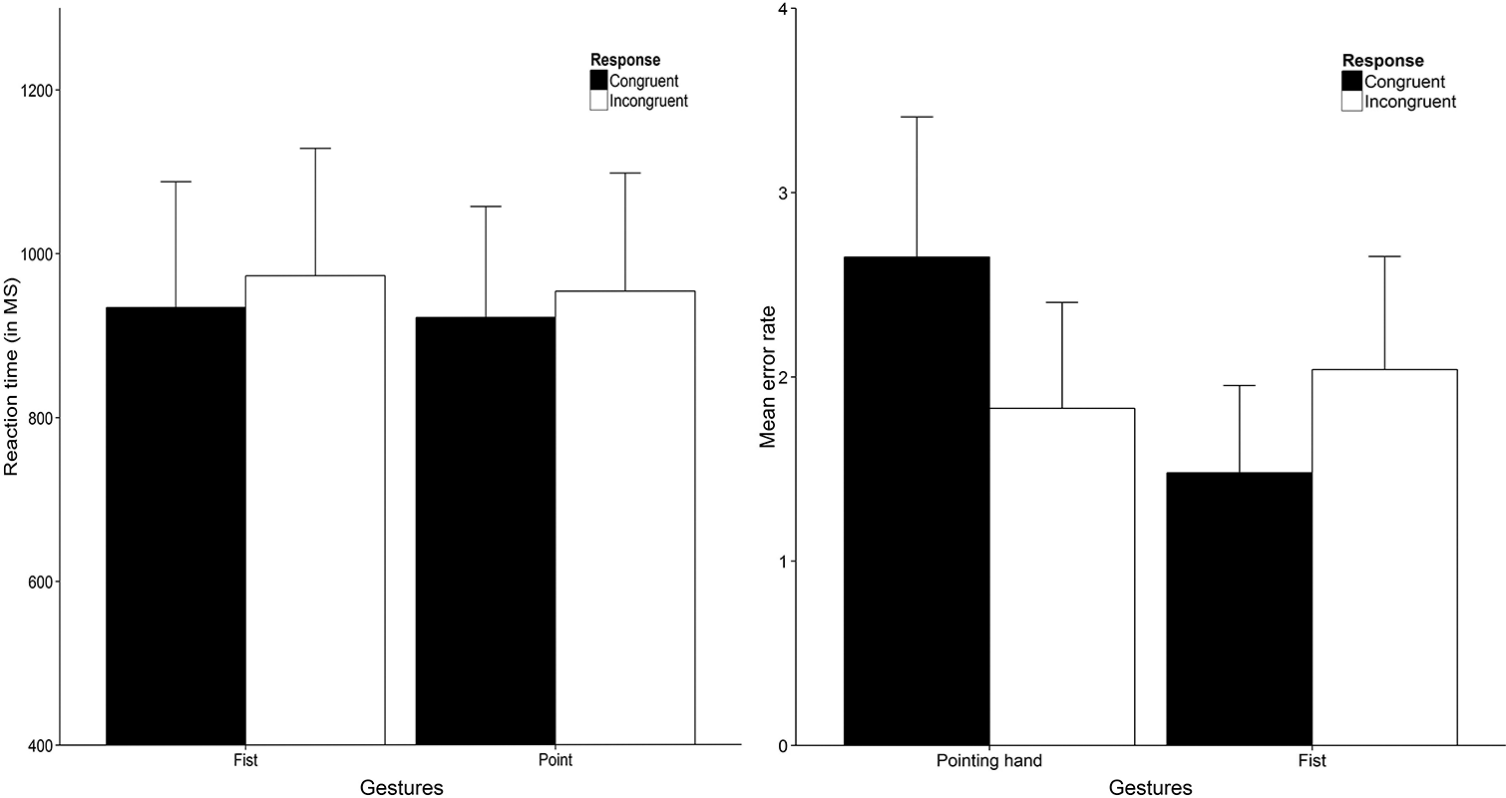
Response data Study 5. Reaction time (left) and response error data (right) for Study 5. Note that a main effect for response was found which is not shown in the figure. Error bars are 1 x SE. * = *p* < .05, ** = *p* < .01, *** = *p* < .001.

For the analysis we included data for 23 participants and ran a 2 x 2 repeated measures design with Hand gesture (fist vs pointing hand) and Hand response (congruent vs incongruent) as within-subject factors (see Fig 9). The analysis revealed a main effect for Hand response in which responses were faster for congruent (*M* = 928, *SD* = 693) relative to incongruent responses (*M* = 963, *SD* = 719) irrespective of the type of Hand gesture, *F*(1, 22) = 4.33, *p* = .049, η^2^_p_ = .16. Given the skewness values results were analyzed using transformed (log) RT values as well. The interpretation of the ANOVA results do not differ between running the 2 x 2 analysis using transformed or untransformed estimates. Untransformed analyses are displayed here. No main effect for Hand gesture or an interaction effect between Hand gesture and Hand response was found. Additionally, no main effects or an interaction between Hand gesture and Hand response was found in the error pattern.

### Discussion

The results of the fifth study suggest that the complementary effect found in Study 1–4 cannot be explained by a more general cueing effect as alternative explanation to the social complementary effect. The results are in fact more in favor of a classical spatial congruency effect given the faster response times for spatially congruent relative to incongruent responses–irrespective of the specific hand gesture type that was presented.

## Re-Analysis across Study 1–4

**Fig 10:**
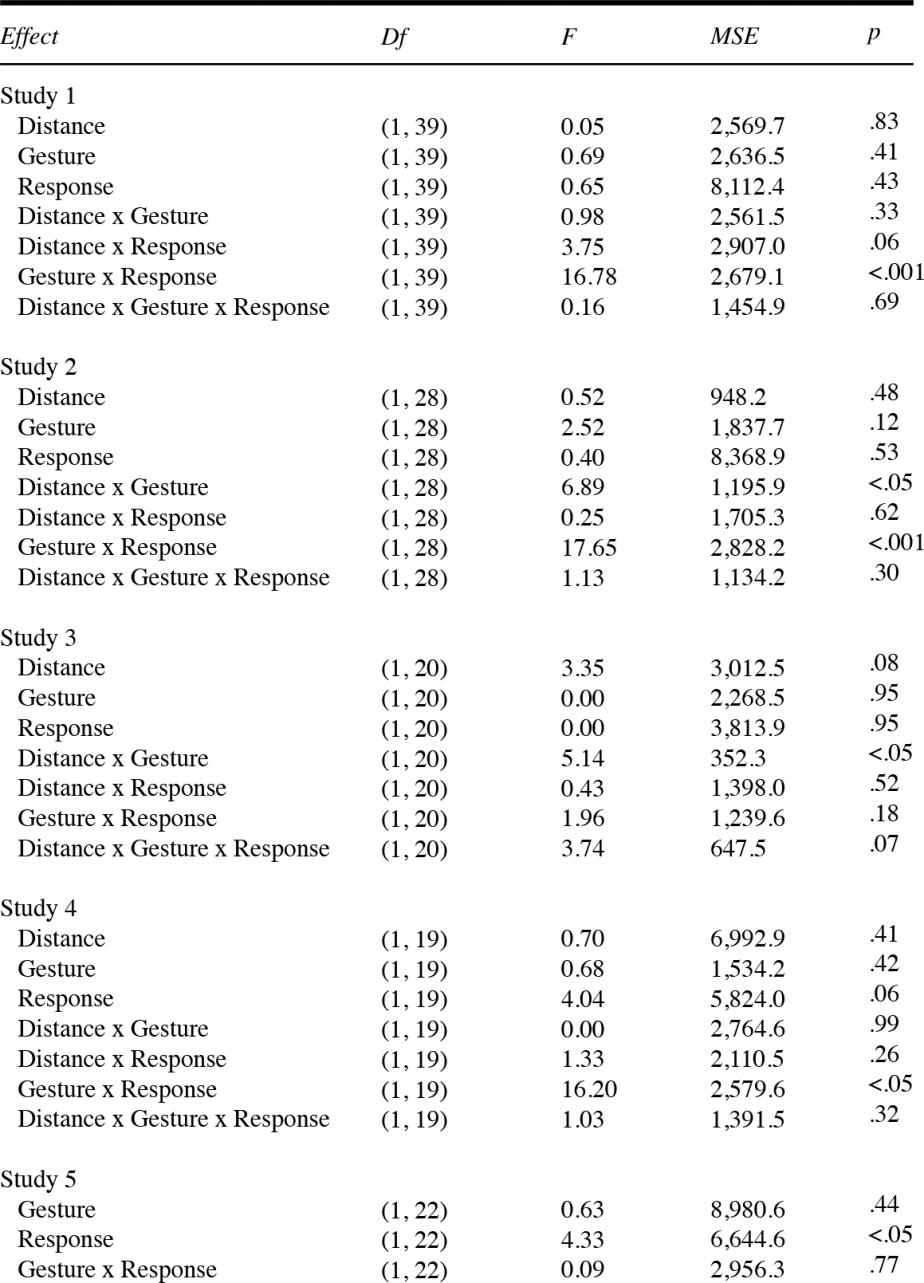
ANOVA results. ANOVA results for reaction time data (studies 1 through 5). Note: The factor Hand gesture is termed Gesture and Hand response termed Response.

**Fig 11:**
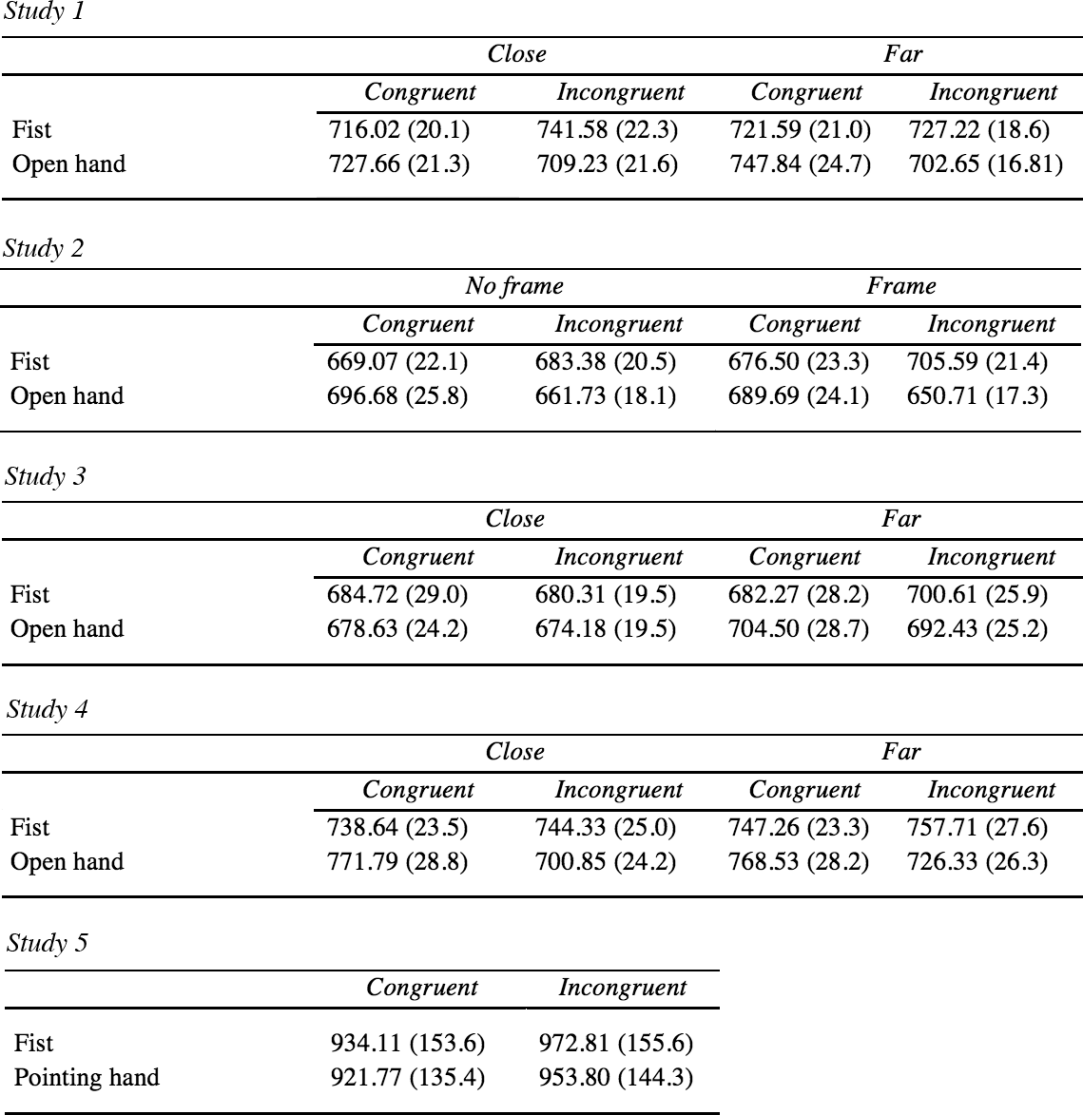
Reaction time results. Mean reaction time results for Study 1 through 5 as a function of distance (or frame) and congruency (columns) for both fist and open hand gestures (+*SE*s within brackets).

Some of the effects observed appeared to be quite stable across the studies described here (i.e., the complementary effect for open hands) but other effects were less consistent (i.e., the effect of distance). Therefore, to get an overview of the combined effects over the first four studies (see Fig 10 and Fig 11) a combined analysis was conducted, with study as a grouping factor. The primary goal of this analysis was to see whether the complementary effect would be stronger in some studies compared to others, if the variability in subjective ratings would affect a sufficiently powered combined sample and whether differences in handedness would modulate the complementary effect. The design for this analysis was a 4 x 2 x 2 x 2 mixed design with Study (Study 1 to 4) as a between participants factor and Distance (close vs far), Hand gesture (fist vs open hand) and Hand response (congruent vs incongruent) as within-subject factors.

A significant main effect for Distance was found, demonstrating faster responses for close (*M* = 706, *SD* = 123) relative to far distance trials (*M* = 712, *SD* = 124), *F*(1, 106) = 3.96, *p* = .049, η^2^_p_ = .04. However, this effect is weak at best considering the large sample size. Additionally, a significant interaction was found between Hand gesture and Hand response, *F*(1, 106) = 45.30, *p* < .001, η^2^_p_ = .30. Interestingly, averaged over studies we found a congruency effect for fist gestures in that faster responses were made in congruent *(M* = 704, *SD* = 122) relative to incongruent response trials (*M* = 719, *SD* = 116), *t*(109) = -2.34, *p* = .021, *d* = -0.22. For open hand gestures, faster responses were made in incongruent (*M* = 690, *SD* = 107) relative to congruent response trials (*M* = 723, *SD* = 132), *t*(109) = 4.76, *p* < .001, *d* = 0.46. No interaction effect between Study and any of the other within-subject factors was found (range: *p* = .082 –*p* = .802). In addition we re-analyzed the raw data using a 3 x SD cutoff for outlier removal given that studies seem to differ in setting the criterion for removal (e.g., Liepelt et al., 2010; [28]) which might affect the robustness of our effects. Across studies 1–4 we found a similar interaction effect between Hand gesture and Hand response, *F*(1, 109) = 42.99, *p* < .001, η^2^_p_ = .28 as well as a main effect for gesture, *F*(1, 109) = 4.44, *p* = .037, η^2^_p_ = .04. None of the other factors reached significance (all *p*s > .05).

Another issue to resolve here is the variability in reachability ratings. Given that the images used in the first four studies were made to reflect settings in which physical contact was either possible or not the subjective ratings indicated that not all participants perceived this to be the case, which might be due to the specific stimulus set we used. This is problematic given that our hypothesis requires participants to correctly identify differences in close and far distance. The subjective estimates however resemble those used in other judgment tasks (e.g., Valdés-Conroy et al., 2014; [11]) even though participants in our studies had to picture themselves in a situation rather than being physically part of it. Additionally, in a separate post-test rating we found that subjects did (more) accurately interpret the pictures if asked to indicate whether the actor was within their personal space or whether extending one’s hand made it possible to touch an extended hand of the actor (see the S1 Appendix). This suggests that the variability in ratings might be due to the specific question we used. In terms of the current re-analysis one solution we used was to look at the role of Reachability in each study separately by splitting participants in groups that did or not did not accurately perceive the distance estimates. However, this might be affected by power issues (insufficient sample sizes) in each individual study. In order to circumvent this problem we added Reachability (averaged across studies) to the across-studies analysis, by putting all participants who successfully perceived our distance/ reachability manipulation (actor in close distance is reachable–actor in far distance is unreachable) into one group (53 participants) and the remaining participants in a second group (57 participants). There was no main effect for Reachability (*F* < 1) nor did inclusion of Reachability affect the interaction between Hand gesture and Hand response, *F*(1, 108) = 48.84, *p* < .001, η^2^_p_ = .31. For more detailed analyses see the S2 Appendix.

Besides Reachability, we added Handedness (left vs right-handed) to the within-participants analysis (averaged over Study 1 to 4). There was no main effect for Handedness (*F* < 1) and although adding handedness did decrease the strength of the Hand gesture by Hand response interaction, it was still significant, *F*(1, 98) = 18.12, *p* < .001, η^2^_p_ = .16. However, the sample sizes (right handed = 113; left handed = 16) were strongly imbalanced so we analyzed data for right and left-handed participants separately. For these analyses we added a within-participants level of Response side (left vs right) to look at differences for the responding hand (e.g., right and left responses to left and right open hand gestures). This resulting analysis was a 2 x 2 x 2 x 2 with Distance (close vs far), Hand gesture (fist vs open hand), Hand response (congruent vs incongruent) and Response side (left vs right). Results showed a strong Hand gesture by Hand response interaction for right-handed participants irrespective of the responding hand, *F*(1, 93) = 40.72, *p* < .001, η^2^_p_ = .31 while for left-handed participants this interaction was around the critical alpha-level of *p* = .05, *F*(1, 11) = 4.83, *p* = .050, η^2^_p_ = .31. However, the sample size does not permit us to make strong conclusions about the group of left-handed participants. Interestingly, for both right and left-handed participants there was no main effect of Response side nor an interaction between Hand gesture, Hand response and Response side (all *F*s < 1), suggesting no modulation of the complementary effect by hand-dominance.

The response error data revealed a main effect for both Hand response, *F*(1, 106) = 8.47. *p* = .004, η^2^_p_ = .07, as well as Hand gesture, *F*(1, 106) = 4.05, *p* = .047, η^2^_p_ = .04 qualified by a two-way interaction between Hand gesture and Hand response, *F*(1, 106) = 36.11, *p* < .001, η^2^_p_ = .25. No difference in the mean error rate for fist gesture trials was found, *t*(109) = -1.90, *p* = .060 but for open hand trials more errors were made across studies in congruent (*M* = 1.25, *SD* = 1.17) relative to incongruent trials (*M* = 0.46, *SD* = 0.73), *t*(109) = 6.46, *p* < .001, *d* = 0.62. When instead of Study, we added Reachability to the within-participants design the Hand gesture by Hand response interaction was still significant (*F*(1, 108) = 43.06, *p* < .001, η^2^_p_ = .29) while Reachability did not interact with any of the remaining within-subject factors.

### Bayesian analysis

Finally, we performed a Bayesian analysis of the full sample (Study 1 to 4) in order to compute the relative evidence in the data in support of the model including the Hand gesture by Hand response interaction compared to the model including the three-way interaction between Distance by Hand gesture by Hand response. Furthermore, we wanted to gauge the relative strength of the simple main effects for fists and open hand gesture trials across distance in order to see how they would fare in comparison to the frequentist analyses. Bayesian analyses provide an opportunity for model comparison (e.g., between two-way and three-way interactions), which allows us to find relative evidence in favor of the two way interaction rather than only refuting the three-way interaction. All analyses were performed using JASP, which is an open source statistical software tool that can be used for both frequentist and Bayesian analyses [49]. First off, we compared the relative fit of an ANOVA model including (Model 1) or excluding (Model 2) the critical 3-way interaction effect between Distance, Hand Gesture and Hand Response (in addition to modeling the main effects and two-way interactions in both models). The relative fit was calculated by dividing the Bayes Factor (BF) of Model 1 by the BF of Model 2 (including the three-way interaction) which produced a BF of ± 5.84 in favor of the simpler model (Model 1; without the three-way interaction). This factor therefore represents the unique contribution of the three-way interaction. Taking these steps was necessary to find the unique variance contribution of a single interaction effect (e.g., the three-way interaction) given that JASP produces models for interaction effects that include all lower level (main) effects.

The same procedure was performed for the two-way interaction between gesture and response. For this interaction we compared the relative fit of a model without the two-way interaction term between gesture and response (Model 3) compared to a model including the two-way interaction term (Model 4; in both models the main effects for gesture and response were included). A BF of ± 97.3 million in favor of Model 4 was found. Subsequently we could infer what the relative fit was of the unique contribution of the two-way interaction term (Hand gesture vs Hand response) compared to the unique contribution of the three-way interaction (Distance vs Hand gesture vs Hand response) by dividing the appropriate Bayes factors by each other. The result suggested that the data was ± 568.3 million times more in favor of the model including the two-way interaction compared to the model including three-way interaction, which counts as sufficiently strong evidence according to interpretation criteria for Bayes Factors [50–51]. Thus, this analysis suggests that our data is best explained by a model describing the interaction between observed gesture and performed response, rather than a model that takes into account the role of distance. This suggests that the effects of observed on performed gestures is quite strong and automatic and occurs irrespective of the distance at which an action is observed.

Additionally, a Bayesian t-test was done comparing the mean RT difference for congruent and incongruent responses to fist gestures and open hand gestures. For fist gestures a BF10 of 1.43 was computed which suggests that support for the alternative hypothesis, as specified by a Cauchy prior distribution d~N(0,.707), compared to the null hypothesis of no effect was weak or inconclusive. This confirms the idea that in larger samples, increasingly larger effects (e.g., t-values) are necessary to produce Bayes factors that favor the alternative hypothesis over the null hypothesis [52]. In contrast, for open hand trials a BF10 of 2504.86 was found suggesting that the data support the alternative hypothesis that there was an effect by a factor of ± 2504 under this specific alternative hypothesis relative to the null hypothesis (which doubled when specifying a directional effect for faster response times in incongruent relative to congruent trials). However, it is important to note that the specified prior distribution is not fully informative (i.e., unspecific) and the results for the fist gesture trials should therefore be interpreted with caution. One way to resolve this is to perform a robustness analysis in which different scaling parameters are used for the prior distribution (instead of 0.707) in order to see how being more or less specific about the predicted distribution affects the Bayes factor estimate. For fist gesture trials, varying the scaling parameter (from 0.5 to 1.5) produced a maximum Bayes factor of 1.85 in favor of the alternative hypothesis (a difference in response times), which falls within the bounds of anecdotal evidence. Even when estimating a directional effect (i.e., faster response times for congruent relative to incongruent trials) with the most narrow distribution here (d~N(0,.5)) the Bayes factor does not exceed 3.66 in favor of the alternative. This suggests weak evidence at best for a congruency effect in fist gesture trials. Given that the chosen ‘default’ prior produces a conservative estimate (biased with respect to small effects) we can be confident with respect to the results for open hand gestures. These results corroborate and extend the findings in the original ANOVA analysis.

## General Discussion

The current paper demonstrates a facilitation effect for performing incongruent responses to observed open hand gestures relative to making congruent responses (i.e., a complementary effect). This effect was only present for open hand gestures but not for intransitive (fist) gestures supporting the idea that the communicative meaning of the hand gesture was driving the facilitation effect. The fifth study supports this conclusion by showing that the complementary effect is not found when replacing the open hand with a pointing hand. The current set of studies therefore replicate the findings in Liepelt et al. (2010; [28]) and Flach et al. (2010; [29]) and extend them by using more environmentally rich stimuli. However, against our predictions, the complementary effect for open hand gestures was found irrespective of the depicted distance in space, either in terms of physical (Study 1) or functional distance (Study 2) between the participant and the person making the gesture. Making space task-relevant, by letting participants directly respond to the spatial context of the perceived gesture, also did not affect the complementary effect. When taking into account ratings of reachability this did not alter the effect suggesting that motor priming (as operationalized here) seems to be driven by the known contingent relationship between a gesture and a response rather than the perceived possibility to perform the response in interaction with another person. However, given the variability in reachability ratings we cannot fully exclude that this unexpected finding might have been related to the use of sub-optimal stimuli. Nevertheless, the results (partly) support the role of sensorimotor learning, in which specific stimuli are associated to actions that do not only reflect spatially or anatomically matching information but learned (social) contingent relations as well (Liepelt et al., 2010; [28]). In contrast to our findings, it has been argued that environmental cues, including interpersonal space or contact, modulate motor priming given the predictive function of these cues in stimulus-response learning [53]. For example, interpersonal space between individuals could be encoded during handshaking so that complementary actions are triggered by open hand gestures specifically when perceived within peripersonal space. Our results do not show any influence of interpersonal space and rather suggest that the affordance provided by perceiving an open hand drives the motor priming effect in an automatic fashion. Interestingly, this seems to be due to the meaning of the gesture rather than being the product of individual experience given that handedness did not modulate the effect (i.e., no preference was detected for right-handed participants to respond with their right vs left hand to open right hands). This seems to be in line with evidence for bimanual responses to complementary actions in right-handers as seen in Sartori, Begliomini, Penozzo, Garolla, and Castiello (2014; [54]).

We did find effects of distance in single studies such as the distance by gesture effect in Study 2 and the distance by gesture effect in Study 3. However, when looking at the Bayesian analysis, only a weak main effect of distance was found, which by computing the Bayes factor of BF01 = 3.40 actually favors the null hypothesis over the model including distance (given a Cauchy prior distribution of d~N(0,.707)). None of the additional interaction effects survived in the overall sample, specifically the theoretically interesting Distance by Hand gesture by Hand response interaction. One reason for the absence of an effect of perceived interpersonal space might have been the nature of our stimulus material. While using a similar paradigm as Liepelt et al. (2010; [28]) in which stimuli were presented using 2D pictures on a computer screen, our third study controlled for the subjective ‘realness’ of the stimulus set and in particular the distance manipulation. Even though the results in this study demonstrated an advantage for hand responses in close distance the follow-up comparisons only showed an effect of distance for open hand gestures irrespective of the type of response (i.e., participants made faster responses to open hand gestures that were presented in close distance).

The absence of the complementary hand effect in the third study may be related to the fact that when presenting the stimuli in 3D, subject’s own hand responses were not visible during the task. The visibility of your own hands might be important given that response-effect associations are partly formed using forward models [55] where a hand movement is accompanied by specific sensory effects. Based on these learned sensorimotor associations, we automatically generate expectations about visual feedback as soon as an action (e.g., hand movement) is initiated (i.e., akin to the ideomotor principle; [56]). The absence of visual feedback from the own hand might thus create a temporary mismatch in the response-effect association for the open hand response, which in turn might have affected response latencies. However, given that the error pattern (albeit small) reflected a tendency to perform complementary actions in response to open hand gestures this remains a speculative explanation.

Furthermore, in the third study the spatial position of gesture cues differed in terms of distance (close vs far) when comparing 3D pictures to the 2D referent pictures. That is, cues were presented more laterally in the close compared to the far distance trials for 3D stimuli due to the physical proximity of the stimuli (screen vs head-mounted display). Specifically, the head-mounted display provides a wide angle view so that the stimulus (actor) is perceived as much larger compared to the 2D pictures on a computer screen (larger in visual degrees), which affects the position of the hand gestures relative to the fixation mark (center of the screen). Previous studies have indicated that congruent motor priming can be modulated by distance in a 3D environment [39]. However, using 3D images to enhance the perception of distance does not seem to be a necessary requirement for the distance effect to be found [10]. This discrepancy between previous findings and our results, relates to a broader discussion about the different potential of affordances in real situations versus pictures depicting real situations [57]. Whereas here we have shown that pictures have the potential to facilitate hand responses as a proxy for physical responses, the role of distance is not evident. Perhaps this is due to a dissociation between processing the information that social actions provide and the possibility to physically respond to these cues, suggesting that pictures might be limited for conveying specific information. Therefore, future studies should experimentally distinguish settings in which participants can realistically interact with the environment from settings where participants passively observe it [34, 58, 59].

Another explanation for the absence of the modulation of space on the complementary effect is that the effect actually reflects the automatic simulation of the partner’s behavior during observation [12], which should not depend on distance. Some studies have shown how in interactive settings, people simulate actions from interacting partners prior to movement [60] and show similar response inhibition patterns in no-go trials even when the no-go rule applies only to the co-acting partner in the task [61]. In the same vein, in the current experiment, participants could be simulating perceived communicative gestures as if they would perform the observed behavior themselves rather than responding to it. This seems plausible given that in our case the observed and performed gesture were similar (matching). However, this idea would go against Liepelt et al. (2010; [28]), van Schie et al. (2008; [25]) as well as Sartori, Bucchioni and Castiello (2013; [62]) in that here responses to complementary requests (in a picture or video presentation task) are interpreted as learned interactive responses rather than responses driven by a first-person simulation of the perceived gesture. In addition, a number of studies have suggested that when imitating observed behavior people tend to imitate movements in a mirrored (specular) fashion rather than in an anatomical similar way [28, 63]. In sum, we think it is unlikely that our findings of a complementary effect for observed open hand gestures simply reflects a process of motor simulation; rather we suggest that the effect reflects the strong and automatic activation of learned associations by responding in a socially prescribed and overlearned fashion.

Besides the success in replicating the complementary effect we failed to replicate the congruency effect for intransitive gestures as demonstrated in Liepelt et al. (2010; [28]; i.e., a mirror congruency effect for fist gestures was expected). Interestingly, though, the meta-analysis across the four studies reported here did show the congruency effect. However, when comparing the congruency effect for fist gestures from the meta-analysis in our study to that found in Liepelt et al. (2010; [28]) it appears that the resulting effect size is roughly four times smaller. This also seemed to affect the Bayesian analysis, which suggested weak or anecdotal evidence for the existence of a congruency effect compared to the frequentist analysis. One of the ways in which our study deviated from this study is that in our study gestures were presented at spatially opposite sides of the screen so that the mirror congruency effect might have been confounded with spatial congruency. Based on Bertenthal, Longo and Kosobud (2006; [48]) we know for example that when dissociated, spatially congruent hand movements in response to intransitive hand gestures are more strongly facilitated than anatomically congruent hand movements. Similarly, for communicative hand gestures, Flach et al. (2010; [29]) found that participants were faster in making hand movements when open hand gestures or arrows were subsequently presented at spatially congruent locations on the screen. However, based on these findings we should have expected a stronger rather than weaker congruency effect for intransitive gestures. A simpler explanation is that our stimuli differ from Liepelt et al. (2010; [28]) in that they depict a full body posture instead of only showing a hand gesture. While van Schie et al. (2008; [25]) as well as Ocampo and Kritikos (2010; [20]) showed an advantage of performing congruent hand responses using similar full-body postures in their studies this effect was driven by the task instruction to either perform congruent or incongruent responses in non-critical trials, something we did not manipulate here. Thus, by adding contextual information, we therefore perhaps strengthened the social information provided in the stimulus (full body vs single hand) which at the same time decreased the salience of the spatial attributes of the stimulus.

So even though some questions remain unsolved, here we have demonstrated and replicated a complementary motor priming effect in response to open hand gestures irrespective of the space in which these gestures are perceived. Note that this effect does not generalize to all complementary actions given that some complementary actions do not require physical interaction (e.g., throwing, catching). On the basis of the current findings we propose that motor priming is primarily a function of stimulus-response coding that does not necessarily correspond with motor priming in real situations where the physical opportunity to respond to objects affects motor responses. Given that effects of space on motor priming have been reported for objects [10], we did not find effects of space on motor priming for hands which could highlight that the social relevance of a target object may automatically necessitate a response to it, which is something for future research to examine. Also, the fact that social and non-social requests differ in terms of the consequences (not responding to a handshake can be a sign of social miscommunication) could be investigated by directly comparing social and non-social target cues in a single design. The current set of studies extends our knowledge of motor priming and demonstrates the limitations of specific information provided in paradigms using pictures (e.g., distance) with respect to the information these pictures are designed to reflect [64].

## Supporting Information

S1 Appendix Reachability ratings. (DOCX)

S2 Appendix Post-test ratings. (DOCX)

S1 Dataset Full data set. (ZIP)
