# Supplementary material for: Complementary Hand Responses Occur in Both Peri- and Extrapersonal Space
Source: PLoS One. 2016 Apr 27;11(4):e0154457. doi: 10.1371/journal.pone.0154457 (PMC4847918; doi:10.1371/journal.pone.0154457)
Supplement: S1 Appendix — (DOCX) [file pone.0154457.s001.docx]

**S1 Appendix. Post-test ratings**

A post-test rating was administered to a separate group of raters. This test was done to further examine the way the pictures were perceived during the experiment and whether other attributes of peripersonal space were accurately perceived in the stimuli used in the task. One such attribute is the notion that peripersonal space requires people to see that objects or other people are within one’s peripersonal space such that extending an arm would allow one to reach and manipulate a target object/ person.

Participants

In total, 46 participants (University of Amsterdam students) were included in the post-test. A ‘catch question’ was included to see if participants would accurately read the instructions. Data for three participants who filled in the catch question (when requested to not do so) was deleted, creating a total of 43 responses. The mean age was 21 years old (range = 18-66) and the sample consisted of 37 female participants and included 35 right-handed participants. All participants received course credit for participation and signed an informed consent before starting the experiment. The test was approved by the University of Amsterdam ethics committee (2015-SP-6354).

Stimuli and Results

For question 1 and 7 a picture was added from Study 1 depicting an actor sitting in either close or far distance making an open hand gesture with a right hand.

|  | **Answers** | | |
| --- | --- | --- | --- |
| **Questions** | **Yes** | **No** | **Unclear** |
| 1. Do you feel it is possible to touch the hand of the person on the opposite side of the table if this person was sitting opposite to the **short end of the table** (see picture below)? | 30 | 11 | 2 |
| For the following questions, imagine you are sitting opposite to the person in the picture (opposite to the **short end of the table**). |  | | |
| 1. Is the person within reach? | 34 | 8 | 1 |
| 1. If you and the person would extend their arms, would you be able to touch each other? | 41 | 2 | 0 |
| 1. Can you shake the person’s hand? | 39 | 2 | 2 |
| 1. Could you understand the person if he/she would say something? | 43 | 0 | 0 |
| 1. Could you catch a ball if this person were to throw one at you? | 41 | 2 | 0 |
| 1. Do you feel it is possible to touch the hand of the person on the opposite side of the table if this person was sitting opposite to **the long end of the table** (see picture below)? | 1 | 40 | 2 |
| For the following questions, imagine you are sitting opposite to the person in the picture (opposite to the **long end of the table**). |  | | |
| 1. Is the person within reach? | 1 | 41 | 1 |
| 1. If you and the person would extend their arms, would you be able to touch each other? | 7 | 34 | 3 |
| 1. Can you shake the person’s hand? | 3 | 37 | 3 |
| 1. Could you understand the person if he/she would say something? | 42 | 1 |  |
| 1. Could you catch a ball if this person were to throw one at you? | 42 | 1 |  |

For all options where unclear was chosen, participants were free to note reasons explaining the clarity of the pictures. With respect to question 2 some participants noted that “the full size of the table was not visible”, or “it is unclear what the size of the table is”. For question 4 one participant noted that “if the person would extend his arm just like in the picture, I would have to move across the table and it would therefore not be possible but if he would extend his full arm it would be possible”. Given the low rate of uncertain choices it can be concluded that the pictures and the distance manipulation presented therein were sufficiently clear. Also, looking at the answers on item 3 and 4, it seems that in terms of peripersonal space, the pictures were seen as depicting settings in which physical contact is possible while in the answers 7, 8, and 9 the majority of participants concluded this was not possible in terms of peripersonal space. One source of confusion could be the length of extension of the arm in the depicted scenes. With respect to the remaining questions, it is clear that in both the close and far distance settings, social interactions that do not require direct physical contact are seen as equally possible (see items 5, 6, 11, and 12).

Taken together, the current findings suggest that (the vast majority of) participants could accurately perceive the pictures as depicting settings in which physical contact would be (im)possible in terms of the interpersonal distance. The items used here might therefore be more specific in referring to the notion of peripersonal space than the original items used in the paper, which might explain the apparent discrepancy between the reachability ratings observed in the main studies and in the post-test.
