# Supplementary material for: Complementary Hand Responses Occur in Both Peri- and Extrapersonal Space
Source: PLoS One. 2016 Apr 27;11(4):e0154457. doi: 10.1371/journal.pone.0154457 (PMC4847918; doi:10.1371/journal.pone.0154457)
Supplement: S2 Appendix — (DOCX) [file pone.0154457.s002.docx]

**S2 Appendix. Reachability ratings**

In study 1-4 we looked at the effect of Reachability, as indicated by the imagined possibility to make physical contact with the perceived actor onscreen (taken from the exit questionnaire), on the main within-subjects design (Distance x Hand gesture x Hand response). We initially looked at Reachability separately for each study before and subsequently conducted a re-analysis of all studies combined.

Reachability in each study was assessed by selecting participants who indicated that it would be possible to touch hands with the actor across from the short end of the table and impossible to touch hands with the actor across from the far end of the table

Study 1

Participants who thought it was possible to touch hands with the actor across from the short end of the table and impossible to touch hands with the actor across from the far end of the table were put in the first group (*n* = 16), the remaining participants were put in the second group (*n* = 24). By adding Reachability to the full 2 x 2 x 2 repeated measures analysis we found a significant interaction effect for Distance and Hand response, *F*(1, 38) = 4.50, *p* = .041, η^2^_p_ = .11. For this interaction no simple main effects reached significance. More importantly, the interaction between Hand gesture and Hand response was still significant when including Reachability in the analysis, *F*(1, 38) = 15.69, *p* < .001, η^2^_p_ = .29. The remaining effects including interactions between Reachability and within participants factors (Distance, Hand gesture and Hand response) were all non-significant (all *F*s < 1). So adding Reachability did not affect the hypothesized three-way interaction between Distance, Hand gesture and Hand response.

Study 2

As in the first study we added Reachability as a between participants factor to see how ratings of the perceived ability to interact would affect response time data. Fifteen participants indicated that it was possible to make physical contact in the no frame trials and impossible in the glass frame trials. All remaining participants were put in the second group. If this variable was added to the analysis we again found an interaction effect for Hand gesture and Hand response, *F*(1, 27) = 17.07, *p* < .001, η^2^_p_ = .39. Also the interaction between Frame and Hand gesture was still significant, *F*(1, 27) = 6.60, *p* = .016, η^2^_p_ = .20. However, Reachability did not interact with any of the remaining variables.

Study 3

In terms of Reachability, one group was formed with twelve participants who indicated it was possible to make physical contact with the actor in close but not far distance trials, the remaining participants were put in the second group. Adding this grouping variable as between participants factor did not affect the Distance by Hand gesture interaction, *F*(1, 19) = 5.63, *p* = .028, η^2^_p_ = .23. No other main and interaction effects were found.

Study 4

Based on the reachability ratings, two groups could be distinguished, including one group of 10 participants who thought it was possible to touch the hands of the actor across from the short end of the table and impossible from the far end of the table. The remaining participants were put in the second group. The interaction effect between Hand gesture and Hand response was still significant when including Reachability in the within-subjects analysis, *F*(1, 18) = 15.35, *p* < .001, η^2^_p_ = .46. There was no main effect of Reachability nor did Reachability interact with the Hand gesture by Hand response interaction (both *F*s < 1).
